# Supplementary material for: Generation and Characterization of Cisplatin-Resistant Oral Squamous Cell Carcinoma Cells Displaying an Epithelial–Mesenchymal Transition Signature
Source: Cells. 2025 Aug 24;14(17):1311. doi: 10.3390/cells14171311 (PMC12427644; doi:10.3390/cells14171311)
Supplement: Supplementary file 1 [file cells-14-01311-s001.zip › File S1.pdf]

File S1. Western blot analysis.

The proteins were extracted from cells using a lysis buffer (10% sucrose, 1% NP-40, 20 mM Tris-HCl, 137 mM NaCl, 10% glycerol, and 2 mM EDTA) containing protease inhibitors (Roche Diagnosis, USA). Total protein (20 μg per sample) was separated by electrophoresis on 10% sodium dodecyl sulfate polyacrylamide gel (SDS-PAGE) under reducing conditions, and they were then transferred to nitrocellulose membranes. Antibodies against TWIST1 (WH0007291M1; Sigma-Aldrich, USA; 1:1000 dilution), E-Cadherin (24E10; Cell Signaling, USA; 1:1000 dilution), anti-Vimentin (D21H3; Cell Signaling, USA; 1:1000 dilution), SNAIL1 (C15D3; Cell Signaling, USA; 1:500 dilution) or monoclonal mouse antibody against β-actin (A-5441; Sigma-Aldrich, USA; 1:10.000 dilution) were incubated for 2 hours on the membranes before being rinsed. The protein bands were detected using an enhanced chemiluminescence system (Merck-Millipore, Germany), and signals were captured with an Alliance 9.7 instrument (UVITEC, UK).
