# Supplementary material for: Generation and Characterization of Cisplatin-Resistant Oral Squamous Cell Carcinoma Cells Displaying an Epithelial–Mesenchymal Transition Signature
Source: Cells. 2025 Aug 24;14(17):1311. doi: 10.3390/cells14171311 (PMC12427644; doi:10.3390/cells14171311)
Supplement: Supplementary file 1 [file cells-14-01311-s001.zip › Table S1.pdf]

Table S1. Sequence of primers used in RT-qPCR.

Gene	Forward primer (5'-3')	Reverse primer (5'-3')
E-cadherin	ACAGCCCCGCCTTATGATT	TCGGAACCGCTTCCTTCA
Vimentin	GGCTCGTCACCTTCGTGAAT	TCAATGTCAAGGGCCATCTTAA
N-cadherin	TGGGAATCCGACGAATGG	GCAGATCGGACCGGATACTG
TWIST1	CGGGAGTCCGCAGTCTTA	CTTGAGGGTCTGAATCTTGCT
SNAIL1	GCGTGTGCTCGGACCTTCT	ATCCTGAGCAGCCGGACTCT
SNAIL2	GGAGCATACAGCCCCATCA	TGGTAGCTGGGCGTGGAA
ZEB1	GCTTTCCCATTCTGGCTCCTA	TCTTGGTCGCCCATTCACA
ZEB2	AAGATAGGTGGCGCGTGTTT	ACTGACGTGTTACGCCTCTTCTAA
MMP-2	GTTCATTTGGCGGACTGT	AGGGTGCTGGCTGAGTAG
MMP-9	CTTTGGACACGCACGAC	CCACCTGGTTCAACTCACT
PPIA	GCTTTGGGTCCAGGAATGG	GTTGTCCACAGTCAGCAATGGT
