# Supplementary material for: Generation and Characterization of Cisplatin-Resistant Oral Squamous Cell Carcinoma Cells Displaying an Epithelial–Mesenchymal Transition Signature
Source: Cells. 2025 Aug 24;14(17):1311. doi: 10.3390/cells14171311 (PMC12427644; doi:10.3390/cells14171311)
Supplement: Supplementary file 1 [file cells-14-01311-s001.zip › Table S2.pdf]

Table S2. Primary antibodies used in immunofluorescence.

Primary Antibody	Dilution	Catalog	Source
TWIST1	1:1000	WH0007291M1	Sigma-Aldrich, USA
E-cadherin	1:1000	24E10	Cell Signaling, USA
Vimentin	1:1000	D21H3	Cell Signaling, USA
N-cadherin	1:1000	SAB5600069	Sigma-Aldrich, USA
