# Supplementary material for: Generation and Characterization of Cisplatin-Resistant Oral Squamous Cell Carcinoma Cells Displaying an Epithelial–Mesenchymal Transition Signature
Source: Cells. 2025 Aug 24;14(17):1311. doi: 10.3390/cells14171311 (PMC12427644; doi:10.3390/cells14171311)
Supplement: Supplementary file 1 [file cells-14-01311-s001.zip › Table S3.pdf]

Table S3A. Gene Ontology (GO) of biological process: terms for HSC-3R upregulated genes.

GOID	GO Term (Biological Process)	% Associated Genes	Associated Genes	Term p-value corrected with Benjamini-Hochberg	Fold Enrichment
GO:0051216	cartilage development	4,27	CSGALNACT1, ZEB1, ZBTB16, WNT5A, COL6A1, TIMP1, BMP6, BMP5	1.74E-6	13.780726256983241
GO:0008284	positive regulation of cell population proliferation	8,55	PTGFR, TSLP, GLP2R, TNC, LIFR, TBX3, BMP6, BMP5, EREG, BTC, PLAC8, S100P, S1PR3, TIMP1, IL7R, FGFR1	5.73E-5	3.486033519553073
GO:0030198	extracellular matrix organization	4,81	CSGALNACT1, ADAMTS15, MMP13, MMP1, COL6A2, COL5A3, PAPLN, SLC39A8, MMP10	1.79E-4	5.735331158975685
GO:0003323	type B pancreatic cell development	2,13	CDH2, WNT5A, BMP6, BMP5	1.93E-4	33.92178770949721
GO:0010467	gene expression	4,27	SLC1A1, COL6A1, KCNQ3, ENPP1, IL7R, TLR4, FGFR1, FBN1	2.96E-4	6.255081421609414
GO:0007411	axon guidance	4,81	SEMA5A, ROBO4, PTPRS, FLRT3, WNT5A, PAPLN, NCAM1, SLIT2, SCN1B	6.16E-4	4.770251396648045
GO:0030539	male genitalia development	2,13	ROR2, TBX3, BMP6, BMP5	7.35E-4	22.049162011173188
GO:0010628	positive regulation of gene expression	7,48	PTGFR, PDE2A, WNT5A, TNC, PTPN22, BMP6, ADAM19, CDH5,	7.43E-4	3.032301258903974

			C1QTNF1, PPARG, VIM, IL7R, TLR4, NTSR1		
GO:0031175	neuron projection development	3,74	FLRT3, MAP1B, TNC, VIM, MAPT, RAB6B, FGFR1	9.189E-4	6.223553793476302
GO:0001501	skeletal system development	3,74	TRPS1, NPR3, TBX3, BMP6, FGFR1, FBN1, BMP5	0.001	5.936312849162011
GO:0007268	chemical synaptic transmission	4,81	DTNA, DLG4, SLC1A1, PMP22, KCNQ3, NRXN2, HTR3A, SYN2, NTSR1	0.001	4.276777114236179
GO:0060907	positive regulation of macrophage cytokine production	2,13	GPRC5B, WNT5A, LAPTM5, TLR4	0.001	17.639329608938546
GO:0007156	homophilic cell adhesion via plasma membrane adhesion molecules	4,27	CDH5, ROBO4, PTPRS, CDH2, PAPLN, PECAM1, NCAM1, NECTIN3	0.001	4.71639829116004
GO:0006954	inflammatory response	6,41	GGT5, IL1RL1, PTGFR, PTGER2, WNT5A, COL6A1, FPR1, S1PR3, SCG2, TLR4, CHST2, BMP6	0.001	3.1349519447165664
GO:0050869	negative regulation of B cell activation	1,60	LAPTM5, FOXJ1, SAMSN1	0.002	41.34217877094972
GO:0001525	angiogenesis	4,81	ROBO4, FAP, MCAM, TIE1, NPR3, SCG2, ARHGAP24, FGFR1, EREG	0.002	3.860748212073126
GO:0007155	cell adhesion	6,95	SEMA5A, RIPOR2, PTPRS, MCAM, TNC, THBS2, CDH2, FAP, COL6A2, COL6A1, NCAM1, CD33, SCN1B	0.002	2.7721383573041725
GO:0007204	positive regulation of cytosolic calcium ion concentration	3,74	PTGFR, C1QTNF1, DLG4, PTGER2, FPR1, S1PR3, CD55	0.002	4.946927374301676

GO:0007416	synapse assembly	2,67	CDH2, FLRT3, MAP1B, NRXN2, MAPT	0.003	8.227299257900441
GO:0050729	positive regulation of inflammatory response	3,20	IL1RL1, GPRC5B, TSLP, PDE2A, WNT5A, TLR4	0.003	6.01340782122905
GO:0010977	negative regulation of neuron projection development	2,67	PTPRS, RTN4RL1, DPYSL3, PMP22, VIM	0.003	7.763789440553937
GO:0043542	endothelial cell migration	2,13	ZEB2, FAP, S100P, SCG2	0.004	12.249534450651769
GO:0010976	positive regulation of neuron projection development	3,20	ATP8A2, DPYSL3, WNT5A, ROR2, SCN1B, FGFR1	0.005	5.334474680122545
GO:0030574	collagen catabolic process	2,13	MRC2, MMP13, MMP1, MMP10	0.005	10.755688785938139
GO:0007275	multicellular organism development	2,67	CDH5, CDH2, TIE1, ROR2, FGFR1	0.006	6.805296917028761
GO:0032722	positive regulation of chemokine production	2,13	IL1RL1, TSLP, WNT5A, TLR4	0.006	10.255424191243343
GO:0045666	positive regulation of neuron differentiation	2,67	GPRC5B, ZEB1, MAP1B, BMP6, FGFR1	0.007	6.335966095164708
GO:0022617	extracellular matrix disassembly	2,13	ADAMTS15, MMP13, MMP1, MMP10	0.008	9.58659217877095
GO:0009410	response to xenobiotic stimulus	4,27	ABCA2, NNMT, CPS1, MAP1B, SLC1A1, COL6A1, CA9, PPARG	0.008	3.4723089781375096
GO:0007165	signal transduction	1,12	IGFBP1, DTNA, TIE1, NPR3, GABRA3, FPR1, NRXN2, STC1, CBLB, ARHGAP24, IL1RL1, DLG4, CHN1, CAPN5, PECAM1, PPARG, TIMP1, ROR2, IL7R, CD33, FBN1	0.008	1.8670661380428908
GO:0032496	response to lipopolysaccharide	3,20	PTGFR, CPS1, PTGER2, COL6A1, PTPN22, TLR4	0.008	4.758811944857522

GO:0014002	astrocyte development	1,60	VIM, ROR2, TLR4	0.010	19.455142951035164
GO:0050829	defense response to Gram-negative bacterium	2,67	TSLP, DMBT1, H2BC11, DEFB1, TLR4	0.010	5.80241105557189
GO:0010951	negative regulation of endopeptidase activity	2,13	SERPINE2, TIMP1, TIMP4, SERPINA5	0.010	8.646730200460071
GO:0071380	cellular response to prostaglandin E stimulus	1,60	GNG2, PTGER2, PPARG	0.011	18.37430167597765
GO:0007507	heart development	3,74	POPDC3, FLRT3, COL6A1, FOXJ1, PPARG, FBN1, STRA6	0.012	3.6401918414672707
GO:0050770	regulation of axonogenesis	1,60	BRSK1, CDH2, CHN1	0.013	16.53687150837989
GO:0043032	positive regulation of macrophage activation	1,60	IL1RL1, WNT5A, TLR4	0.013	16.53687150837989
GO:0009612	response to mechanical stimulus	2,13904E+14	MAP1B, COL6A1, TNC, PPARG	0.014	7.736548074095854
GO:0061028	establishment of endothelial barrier	1,60428E+16	ROBO4, PDE2A, PECAM1	0.015	15.749401436552274
GO:0030282	bone mineralization	2,13904E+14	MMP13, COL6A1, ENPP1, ROR2	0.015	7.603159314197651
GO:0071676	negative regulation of mononuclear cell migration	1,06952E+14	SLIT2, BMP5	0.017	110.24581005586593
GO:0010596	negative regulation of endothelial cell migration	1,60428E+16	STC1, SLIT2, DCN	0.019	13.78072625698324
GO:0070593	dendrite self-avoidance	1,60428E+16	ROBO4, PTPRS, PAPLN	0.022	12.720670391061452
GO:0071560	cellular response to transforming growth factor beta stimulus	2,13904E+14	ZEB1, PDE2A, WNT5A, FBN1	0.024	6.391061452513966

GO:0090263	positive regulation of canonical Wnt signaling pathway	2,6738E+16	SEMA5A, GPRC5B, ZEB2, DAAM2, TBL1X	0.025	4.481536994140891
GO:0050860	negative regulation of T cell receptor signaling pathway	1,60428E+16	LAPTM5, CBLB, PTPN22	0.026	11.812051077414205
GO:0007269	neurotransmitter secretion	1,60428E+16	BRSK1, NRXN2, SYN2	0.026	11.812051077414205
GO:0036293	response to decreased oxygen levels	1,06952E+14	SLC1A1, COL6A1	0.026	73.49720670391062
GO:2000860	positive regulation of aldosterone secretion	1,06952E+14	C1QTNF1, BMP6	0.026	73.49720670391062
GO:0097118	neuroligin clustering involved in postsynaptic membrane assembly	1,06952E+14	CDH2, NRXN2	0.026	73.49720670391062
GO:0001938	positive regulation of endothelial cell proliferation	2,13	SEMA5A, WNT5A, SCG2, BMP6	0.027	6.1247672253258845
GO:0043410	positive regulation of MAPK cascade	3,20	C1QTNF1, GPR37, CDH2, WNT5A, LAPTM5, FGFR1	0.028	3.5184832996552955
GO:0006508	proteolysis	5,88	GGT5, ADAMTS15, MMP13, FAP, MMP1, PAPPA, CAPN5, PAPLN, HTRA1, TBL1X, MMP10	0.029	2.188996228546074
GO:0050808	synapse organization	2,13	PTPRS, SLC1A1, NCAM1, MAPT	0.029	5.9592329759927525
GO:0071260	cellular response to mechanical stimulus	2,13	RIPOR2, PDE2A, TLR4, BMP6	0.032	5.727055067837191
GO:0001958	endochondral ossification	1,60	CSGALNACT1, MMP13, BMP6	0.035	10.022346368715084
GO:0051491	positive regulation of filopodium assembly	1,60	RIPOR2, DOCK11, DPYSL3	0.035	10.022346368715084
GO:0048469	cell maturation	1,60	FOXJ1, PPARG, FGFR1	0.035	10.022346368715084

GO:0038111	interleukin-7-mediated signaling pathway	1,06	TSLP, IL7R	0.035	55.122905027932966
GO:1902474	positive regulation of protein localization to synapse	1,06	WNT5A, MAPT	0.035	55.122905027932966
GO:0090258	negative regulation of mitochondrial fission	1,06	PPARG, MAPT	0.035	55.122905027932966
GO:0035633	maintenance of blood-brain barrier	1,60	CDH5, SLC1A1, PECAM1	0.037	9.727571475517582
GO:0048678	response to axon injury	1,60	FLRT3, DPYSL3, SLC1A1	0.039	9.449640861931364
GO:0006882	intracellular zinc ion homeostasis	1,60	SLC1A1, SLC39A8, MT1X	0.039	9.449640861931364
GO:0007389	pattern specification process	1,60	ZEB1, FOXJ1, BMP5	0.039	9.449640861931364
GO:0006955	immune response	5,34	IL1RL1, ENPP2, PECAM1, ENPP1, CBLB, DEFB1, IL7R, TLR4, IFI44L, BMP6	0.039	2.183085347640909
GO:0030509	BMP signaling pathway	2,13	WNT5A, PPARG, BMP6, BMP5	0.042	5.127712095621671
GO:0001890	placenta development	1,60	ADAM19, HTRA1, PPARG	0.043	8.93884946398913
GO:0048752	semicircular canal morphogenesis	1,06	ZEB1, TBX3	0.0442	44.098324022346375
GO:0070779	D-aspartate import across plasma membrane	1,06	SLC1A1, NTSR1	0.044	44.098324022346375
GO:0097325	melanocyte proliferation	1,06	FAP, WNT5A	0.044	44.098324022346375
GO:0071799	cellular response to prostaglandin D stimulus	1,06	PTGFR, TNC	0.044	44.098324022346375

GO:0033363	secretory granule organization	1,06	SRGN, SERPINE2	0.044	44.098324022346375
GO:0006869	lipid transport	2,13	ABCA2, OSBPL6, ABCA5, SERPINA5	0.045	5.011173184357542
GO:0098609	cell-cell adhesion	3,20	CDH5, ROBO4, CDH2, DLG4, PECAM1, CD33	0.045	3.0766272573730022
GO:2000352	negative regulation of endothelial cell apoptotic process	1,60	SEMA5A, CDH5, SCG2	0.046	8.703616583357835

Table S3B. Gene Ontology (GO) of molecular function: terms for HSC-3R upregulated genes.

GOID (Molecular Function)	GO Term (Molecular Function)	% Associated Genes	Associated Genes	Term p-value corrected with Benjamini-Hochberg	Fold Enrichment
GO:0005518	collagen binding	4,28	MRC2, C1QTNF1, MMP13, PCOLCE2, LUM, COL6A2, COL5A3, COL6A1	4.18E-6	12.098080689385037
GO:0008201	heparin binding	5,88	ADAMTS15, PTPRS, PCOLCE2, SERPINE2, RTN4RL1, COL5A3, SLIT2, THBS2, SERPINA5, FGFR1, FBN1	1.37E-5	6.105347901092583
GO:0004222	metalloendopeptidase activity	3,74	ADAM19, ADAMTS15, MMP13, MMP1, PAPPA, PAPLN, MMP10	8.24E-4	6.351492361927145
GO:0035374	chondroitin sulfate binding	1,60	PTPRS, RTN4RL1, DPYSL3	0.004696632689531768	28.45798525798526
GO:0070700	BMP receptor binding	1,60	CDH5, BMP6, BMP5	0.005600772524545212	26.08648648648649
GO:0004175	endopeptidase activity	2,67	ADAMTS15, MMP13, CPS1, FAP, MMP1	0.005736872971801566	6.956396396396397
GO:0098632	cell-cell adhesion mediator activity	2,14	ROBO4, PTPRS, PAPLN, NCAM1	0.00901312591073749	9.275195195195197
GO:0019903	protein phosphatase binding	2,67	CDH5, CDH2, DLG4, PPARG, CD33	0.010402048501706356	5.862131794716065
GO:0008270	zinc ion binding	9,09	DTNA, MMP1, PDE2A, CBLB, MT1X, MMP10, RTL3, ADAMTS15, MMP13, ZEB1,	0.011942768150257698	1.9797779922779923

			PAPPA, TRPS1, ENPP2, ENPP1, CA9, PPARG, TIMP1		
GO:0042277	peptide binding	2,14	TMEM158, GPR37, NPR3, PPARG	0.01555122352067485	7.58879606879607
GO:0036221	UTP diphosphatase activity	1,07	ENPP1, ASMTL	0.018973042267361854	104.34594594594596
GO:0004867	serine-type endopeptidase inhibitor activity	2,67	PCSK1N, SERPINE2, PAPLN, TFPI, SERPINA5	0.019882417786890278	4.830830830830831
GO:0005539	glycosaminoglycan binding	1,60	SERPINE2, DCN, SERPINA5	0.025277252331996954	12.03991683991684
GO:0005201	extracellular matrix structural constituent	2,67	LUM, COL5A3, TNC, THBS2, FBN1	0.025769044551493883	4.459228459228459
GO:0005044	scavenger receptor activity	1,60	DMBT1, ENPP2, ENPP1	0.029037824242408108	11.17992277992278
GO:0017124	SH3 domain binding	2,67	ADAM19, DPYSL3, CBLB, PTPN22, MAPT	0.03260120747125475	4.140712140712141
GO:0005125	cytokine activity	3,21	TSLP, WNT5A, TIMP1, SCG2, BMP6, BMP5	0.03877437356128237	3.210644490644491
GO:0004252	serine-type endopeptidase activity	3,21	MMP13, DMBT1, FAP, MMP1, HTRA1, MMP10	0.04137961925403458	3.161998361998362
GO:0008146	sulfotransferase activity	1,60	SULT1C2, SULT1B1, CHST2	0.04377710652298148	8.943938223938224
GO:0045545	syndecan binding	1,07	SEMA5A, TNC	0.04676335420736293	41.73837837837838
GO:0004528	phosphodiesterase I activity	1,07	ENPP2, ENPP1	0.04676335420736293	41.73837837837838
GO:0097109	neuroligin family protein binding	1,07	DLG4, NRXN2	0.04676335420736293	41.73837837837838
GO:0005509	calcium ion binding	6,95	PROS1, CBLB, THBS2, CDH5, MMP13, CDH2, CPS1, ENPP2,	0.05397834893509679	1.818360988334179

			ENPP1, S100P, MAN1A1, SLIT2, FBN1		
GO:0016791	phosphatase activity	1,60	PXYLP1, ENPP1, PTPN22	0.06325982558502229	7.2799497171590195
GO:0038023	signaling receptor activity	3,21	MRC2, ROBO4, RTN4RL1, TLR4, CD33, STRA6	0.0645577560885545	2.7825585585585584
GO:0047429	nucleoside triphosphate diphosphatase activity	1,07	ENPP1, ASMTL	0.06485381673770617	29.813127413127415
GO:0042802	identical protein binding	1,28	RIPOR2, ZBTB16, SLC1A1, PDE2A, HTRA1, STC1, DEFB1, SORBS2, HTR3A, SYN2, C1QTNF1, CDH2, FAP, DPYSL3, PPARG, VIM, MAPT, SLIT2, TBL1X, NECTIN3, TLR4, NTSR1, FGFR1, FBN1	0.06685296928501713	1.4417401857816365
GO:0004896	cytokine receptor activity	1,60	IL1RL1, LIFR, IL7R	0.0685132812590464	6.956396396396397
GO:0004866	endopeptidase inhibitor activity	1,60	PCSK1N, PROS1, TFPI	0.0685132812590464	6.956396396396397
GO:0008083	growth factor activity	2,67	BTC, TIMP1, BMP6, EREG, BMP5	0.07301654746606681	3.1619983619983625
GO:0008376	acetylgalactosaminyltransferase activity	1,07	CSGALNACT1, B4GALNT4	0.07377058606500966	26.08648648648649
GO:0030020	extracellular matrix structural constituent conferring tensile strength	1,60	COL6A2, COL5A3, COL6A1	0.09376375087402188	5.796996996996998

Table S3C. Gene Ontology (GO) of cellular component: terms for HSC-3R upregulated genes.

GOID (Cellular Component)	GO Term (Cellular Component)	% Associated Genes	Associated Genes	Term p-value corrected with Benjamini-Hochberg	Fold Enrichment
GO:0005615	extracellular space	2,78	SERPINE2, PROS1, TNC, AKR1B1, DEFB1, SERPINA5, C1QTNF1, DMBT1, DPYSL3, ENPP2, ENPP1, TIMP1, TIMP4, IGFBP1, SRGN, MMP1, WNT5A, MMP10, DCN, EREG, BTC, GPRC5B, MMP13, RTN4RL1, COL6A2, PAPPA, H2BC11, PECAM1, SCG2, TLR4, KRT81, PCOLCE2, PAPLN, HTRA1, STC1, KRT86, TFPI, ADAMTS15, PCSK1N, FLRT3, SLIT2, CD55, MGAM, TSLP, LUM, SUSD2, MCAM, BMP6, BMP5, FAP, COL5A3, FBN1	3.03E-12	2.8878212201379934
GO:0005576	extracellular region	2,51	PTGFR, PCOLCE2, SERPINE2, PROS1, PAPLN, HTRA1, TNC, DEFB1, THBS2, TFPI, SERPINA5, PLAC8, IL1RL1, C1QTNF1, DMBT1, FLRT3, ENPP1, NCAM1, SLIT2, TIMP1, CD55, SCN1B, IGFBP1, SRGN, TSLP, MMP1, LUM, MCAM, WNT5A, MMP10, DCN, EREG, BTC, MMP13, RTN4RL1,	4.12E-9	2.52566731414899

			COL6A2, COL5A3, PAPPA, COL6A1, SELENOP, HHIPL2, S100P, MAPT, IL7R, PNLIPRP3, FGFR1, FBN1		
GO:0005886	plasma membrane	4,49	SEMA5A, ROBO4, PTPRS, ATP8A2, PROS1, CBLB, CDH5, C1QTNF1, DMBT1, CDH2, SLC16A6, ENPP2, ENPP1, CA9, SLC39A8, STK32A, CD33, SCN1B, GPR37, WNT5A, GLP2R, LIFR, HTR3A, SYN2, EREG, ADAM19, BTC, GPRC5B, SLC7A7, RTN4RL1, MAP1B, H2BC11, PECAM1, KCNQ3, MAPT, ROR2, TLR4, GRAMD1B, RGS17, PTGFR, SLC43A2, PTGER2, NPR3, PAPLN, SLC1A1, HTRA1, FPR1, NRXN2, TFPI, IL1RL1, GNG2, FLRT3, NCAM1, S1PR3, SLIT2, NTSR1, CD55, STRA6, GGT5, ABCA2, MGAM, DTNA, OSBPL6, ABCA5, KCNJ12, TIE1, SUSD2, MCAM, PDE2A, GABRA3, LAPTM5, SORBS2, ENTREP1, FAP, CPS1, DLG4, PMP22, VIM, RGL1, EVI2B, IL7R, NECTIN3, MYO1G, FGFR1	6.21E-8	1.6959633453195686
GO:0031012	extracellular matrix	7,48	MMP1, LUM, PAPLN, WNT5A, MMP10, ADAMTS15, MMP13,	2.23E-7	6.585990990990991

			DMBT1, RTN4RL1, FLRT3, TIMP1, TLR4, TIMP4, FBN1		
GO:0062023	collagen-containing extracellular matrix	9,09	PCOLCE2, SERPINE2, LUM, WNT5A, TNC, HTRA1, THBS2, DCN, SERPINA5, ADAM19, ADAMTS15, CDH2, COL6A2, COL5A3, COL6A1, NCAM1, FBN1	7.09E-7	4.70427927927928
GO:0009986	cell surface	1,06	GPR37, SLC1A1, WNT5A, TFPI, ADAMTS15, CDH5, GPRC5B, CDH2, FAP, DMBT1, RTN4RL1, CAPN5, KCNQ3, ENPP1, NCAM1, ROR2, TLR4, CD55, CD33, NTSR1	5.88E-6	3.4369163684232182
GO:0005581	collagen trimer	3,74	C1QTNF1, PCOLCE2, MMP1, COL6A2, COL5A3, COL6A1, TIMP1	2.59E-4	7.903189189189189
GO:0005925	focal adhesion	6,95	NEBL, MCAM, TNC, DIXDC1, SORBS2, ARHGAP24, MRC2, IL1RL1, CDH2, FAP, FLRT3, CAPN5, VIM	4.97E-4	3.374103758931345
GO:0045202	synapse	7,48	RGS17, DTNA, GPR37, SLC1A1, GABRA3, SORBS2, HTR3A, SYN2, DLG4, MAP1B, DPYSL3, CAPN5, PMP22, KCNQ3	5.59E-4	3.12997591651057
GO:0043235	receptor complex	4,81	GPRC5B, PTPRS, GPR37, TIE1, LIFR, PPARG, ROR2, TLR4, FGFR1	8.77E-4	4.516108108108108
GO:0070062	extracellular exosome	1,87	SEMA5A, ROBO4, PTPRS, PROS1, NPR3, SLC1A1, HTRA1,	0.001	1.7500418930888375

			AKR1B1, DEFB1, SERPINA5, GNG2, PCBP3, DMBT1, CAPN5, MAN1A1, SLIT2, TIMP1, CD55, MGAM, LUM, NEBL, SUSD2, WNT5A, LIFR, EML5, GPRC5B, RTN4RL1, DAAM2, COL6A2, COL6A1, SELENOP, PECAM1, S100P, VIM, MYO1G		
GO:0005788	endoplasmic reticulum lumen	5,34	IGFBP1, CDH2, COL6A2, COL5A3, WNT5A, COL6A1, TNC, TIMP1, SCG2, FBN1	0.001	3.6303119840097335
GO:0030424	axon	5,88	ROBO4, PTPRS, DTNA, MAP1B, SLC1A1, PAPLN, NCAM1, VIM, MAPT, NECTIN3, SCN1B	0.002	3.26823613086771
GO:0030669	clathrin-coated endocytic vesicle membrane	2,67	BTC, WNT5A, ROR2, IL7R, EREG	0.003	7.950894556528359
GO:0030027	lamellipodium	3,74	ABLIM3, CDH2, FAP, DPYSL3, SORBS2, ARHGEF6, MYO1G	0.007	4.052917532917533
GO:0031091	platelet alpha granule	1,60	SERPINE2, THBS2, SERPINA5	0.008	21.16925675675676
GO:0016323	basolateral plasma membrane	4,27	SLC7A7, SLC43A2, CDH2, DLG4, SLC16A6, ENPP1, CA9, SLC39A8	0.009	3.3577011956194114
GO:0005604	basement membrane	2,67	COL6A1, TNC, TIMP1, THBS2, FBN1	0.010	5.819726943438284
GO:0045121	membrane raft	3,74	RTN4RL1, SLC1A1, PECAM1, CBLB, MAPT, CD55, NTSR1	0.011	3.745587293454592
GO:0043204~perikaryon		3,20	PTPRS, RTN4RL1, MAP1B, SLC1A1, SCN1B, NTSR1	0.013	4.260479347271801

GO:0009897	external side of plasma membrane	5,34	IL1RL1, CDH5, MCAM, PECAM1, LIFR, NCAM1, IL7R, TLR4, CD33, SERPINA5	0.0139	2.6317646317646317
GO:0042383	sarcolemma	2,67	POPDC3, DTNA, CDH2, COL6A2, COL6A1	0.014	5.275827229098258
GO:0005796	Golgi lumen	2,67	LUM, PROS1, WNT5A, DEFB1, DCN	0.014	5.275827229098258
GO:0016020	membrane	3,10	SEMA5A, ROBO4, TNC, DEFB1, SERPINA5, MRC2, CDH5, DMBT1, SLC16A6, ENPP2, ENPP1, CA9, MAN1A1, SLC39A8, ACSL1, WNT5A, GLP2R, HTR3A, ADAM19, BTC, SLC7A7, MMP13, PAPPA, COL6A1, SELENOP, MAPT, ROR2, TLR4, TMEM45A, GRAMD1B, POPDC3, PRUNE2, NPR3, SLC1A1, FPR1, NRXN2, TMTC1, CYB5R2, GNG2, NCAM1, SLIT2, STRA6, ABCA2, MGAM, DTNA, ABCA5, KCNJ12, TIE1, MCAM, ENTREP1, TMEM158, FAP, PMP22, EVI2B, NECTIN3, MYO1G, FBN1, FGFR1	0.020	1.2972180579946033
GO:0030054	cell junction	3,20	CDH5, BRSK1, DTNA, CDH2, FLRT3, DLG4	0.025	3.6225466107819053
GO:0043005	neuron projection	4,27	RGS17, CDH2, DLG4, SLC1A1, GABRA3, PAPLN, HTR3A, MAPT	0.026	2.753724456163481

GO:0005911	cell-cell junction	3,20	CDH5, CDH2, FLRT3, PECAM1, NECTIN3, ARHGEF6	0.028	3.5099285814311725
GO:0032991	protein-containing complex	6,41	DTNA, CPS1, ZBTB16, TRPS1, COL6A2, NPR3, COL6A1, PECAM1, LAPTM5, NRXN2, SERPINA5, STRA6	0.040	1.9807491702228543
GO:0001725	stress fiber	2,13	ABLIM3, NEBL, LIMCH1, DIXDC1	0.044	5.074278773155178

Table S3D. KEGG terms for the dataset of differentially expressed genes comparing HSC-3R with HSC-3P cell lines: terms for HSC-3R upregulated genes.

KEGGID	KEGG Term	% Associated Genes	Associated Genes	Term p-value corrected with Benjamini-Hochberg	Fold Enrichment
hsa04820	Cytoskeleton in muscle cells	5,34	DTNA, NEBL, DAAM2, COL6A2, COL5A3, COL6A1, VIM, THBS2, DCN, FBN1	0.001	3.528097062579821
hsa04514	Cell adhesion molecules	3,74	CDH5, PTPRS, CDH2, PECAM1, NRXN2, NCAM1, NECTIN3	0.011	3.649445623967917
hsa04610	Complement and coagulation cascades	2,67	SERPINE2, PROS1, TFPI, CD55, SERPINA5	0.021	4.6506734006734005
hsa04151	PI3K-Akt signaling pathway	5,34	GNG2, PPP2R2C, COL6A2, COL6A1, TNC, THBS2, IL7R, TLR4, FGFR1, EREG	0.031	2.26110087988541
hsa04974	Protein digestion and absorption	2,67	SLC7A7, COL6A2, COL5A3, SLC1A1, COL6A1	0.038	3.8977072310405645
hsa00760	Nicotinate and nicotinamide metabolism	1,60	NNMT, ENPP1, NT5M	0.077	6.461988304093566
hsa04512	ECM-receptor interaction	2,13	COL6A2, COL6A1, TNC, THBS2	0.092	3.6787349146899704
