# Supplementary material for: Generation and Characterization of Cisplatin-Resistant Oral Squamous Cell Carcinoma Cells Displaying an Epithelial–Mesenchymal Transition Signature
Source: Cells. 2025 Aug 24;14(17):1311. doi: 10.3390/cells14171311 (PMC12427644; doi:10.3390/cells14171311)
Supplement: Supplementary file 1 [file cells-14-01311-s001.zip › Table S4.pdf]

Table S4A. Gene Ontology (GO) of biological process: terms for HSC-3R downregulated genes.

GOID (Biological Process)	GO Term (Biological Process)	% Associated Genes	Associated Genes	Term p-value corrected with Benjamini-Hochberg	Fold Enrichment
GO:0098609	cell-cell adhesion	5,08	DLG5, PDPN, CNTN1, PKP1, FGFR2, MPZL2	0.006	5.006511627906976
GO:0001525	angiogenesis	5,08475E+15	ARHGAP22, SEMA4A, ANPEP, PDGFA, FGFR2, EPHB3	0.014	4.1883268482490275
GO:0032956	regulation of actin cytoskeleton organization	3,38	PDGFA, GRHL3, FZD10, RHOD	0.016	7.475
GO:0030335	positive regulation of cell migration	5,084	SEMA4A, CLDN4, PDPN, MYADM, PDGFA, RHOD	0.021	3.7505226480836233
GO:0006635	fatty acid beta-oxidation	2,54	MTLN, SLC27A2, ACAT2	0.028	11.212499999999999
GO:0051781	positive regulation of cell division	2,54	MACC1, PDGFA, FGFR2	0.030	10.983673469387755
GO:0061304	retinal blood vessel morphogenesis	1,69	COL4A1, LAMA1	0.038	51.25714285714285
GO:0060174	limb bud formation	1,69	ZNF219, FGFR2	0.048	39.86666666666667
GO:0009611	response to wounding	2,54	SOX2, TMPRSS4, PDGFA	0.051	8.154545454545454

GO:0060445	branching involved in salivary gland morphogenesis	1,69	LAMA1, FGFR2	0.059	32.61818181818182
GO:0007155	cell adhesion	5,93	CLDN4, MUC16, LAMA1, PDPN, CNTN1, PKP1, NPNT	0.066	2.4290135396518373
GO:0001843	neural tube closure	2,54	VANGL2, COBL, GRHL3	0.070	6.812658227848101
GO:0030198	extracellular matrix organization	3,38	COL4A1, MMP17, VWA1, NPNT	0.070	4.147976878612717
GO:0048546	digestive tract morphogenesis	1,69	SFRP5, EPHB3	0.079	23.92
GO:0045176	apical protein localization	1,69	VANGL2, DLG5	0.079	23.92
GO:0001736	establishment of planar polarity	1,69	VANGL2, GRHL3	0.084	22.425
GO:0060441	epithelial tube branching involved in lung morphogenesis	1,69	LAMA1, DLG5	0.089	21.105882352941176
GO:0003382	epithelial cell morphogenesis	1,69	RAB25, POF1B	0.094	19.933333333333334

Table S4B. Gene Ontology (GO) of molecular function: terms for HSC-3R downregulated genes.

GOID (Molecular Function)	GO Term (Molecular Function)	% Associated Genes	Associated Genes	Term p-value corrected with Benjamini-Hochberg	Fold Enrichment
GO:0005201	extracellular matrix structural constituent	4,23	MFAP5, COL4A1, LAMA1, VWA1, NPNT	0.004	7.300506769533318
GO:0004947	bradykinin receptor activity	1,69	BDKRB2, BDKRB1	0.011	170.83185840707966
GO:0017147	Wnt-protein binding	2,54	SFRP5, APCDD1L, FZD10	0.020	13.486725663716815
GO:0019215	intermediate filament binding	1,69	SYNM, PKP1	0.034	56.94395280235988
GO:0008092	cytoskeletal protein binding	2,54	FARP1, DLG5, FLNC	0.037	9.669727834362998
GO:0005544	calcium-dependent phospholipid binding	2,54	ANXA11, SYT8, SYTL3	0.040	9.31810136765889

Table S4C. Gene Ontology (GO) of cellular component: terms for HSC-3R downregulated genes.

GOID (Cellular Component)	GO Term (Cellular Component)	% Associated Genes	Associated Genes	Term p-value corrected with Benjamini-Hochberg	Fold Enrichment
GO:0070062	extracellular exosome	2,28	LAD1, TACSTD2, NDRG2, ACAT2, TGM1, RAB25, ANPEP, COBLL1, SNCG, MYH14, S100A14, DUOX2, PROM2, GBP6, GGT6, MUC16, GALNT3, ANXA11, A2ML1, NPNT, ACTA2, C1ORF116, CNTN1, RAB19, VWA1, SLC27A2, ZNF114	3.02E-12	2.8878212201379934
GO:0005604	basement membrane	5,084	ACTA2, LAD1, COL4A1, LAMA1, VWA1, NPNT	4.12E-9	2.52566731414899
GO:0031410	cytoplasmic vesicle	6,77	GBP6, CADPS2, ADGRF1, RAB25, PDPN, MAPK15, FGFR2, PROM2	6.21E-8	1.6959633453195686
GO:0005886	plasma membrane	3,89	TMPRSS4, FZD10, BEST1, TGM1, PRRG4, RAB25, MARVELD1, DUOXA1, ANPEP, PDPN, BDKRB2, BDKRB1, FLNC, DUOX2, CACNG4, GPR158, EPHB3, MPZL2, PROM2, SH3D19, SEMA4A, ABCC2, MUC16, TMEM30B, SLC6A15, APCDD1L, COBL, ANXA11, SLC6A11, RHOD, SYT8, SYTL3, CLDN4, C1ORF116, VANGL2, ADGRF1,	2.23E-7	6.585990990990991

			USH1G, DLG5, MMP17, MYADM, CNTN1, PKP1, RAB19, LTB, SLC27A2, FGFR2		
GO:0005615	extracellular space	1,69	LAMA1, FST, TMPRSS4, TACSTD2, PDGFA, A2ML1, PRSS56, PRRG4, ACTA2, GAL, IL36B, COL4A1, ANPEP, ALOX5, SFRP5, MMP17, VWA1, IL36RN, LTB, S100A14	7.09E-7	4.70427927927928
GO:0030485	smooth muscle contractile fiber	1,69	ACTA2, NPNT	5.88E-6	3.4369163684232182
GO:0005911	cell-cell junction	4,23	CLDN4, VANGL2, LAMA1, MYADM, MAPK15	2.58E-4	7.903189189189189
GO:0042995	cell projection	4,23	SH3PXD2A, PDPN, SLC6A11, GPR158, PROM2	4.97E-4	3.374103758931345
GO:0062023	collagen-containing extracellular matrix	5,93	MFAP5, COL4A1, LAMA1, ANXA11, VWA1, NPNT, FGFR2	5.59E-4	3.12997591651057
GO:0070161	anchoring junction	2,54	SH3PXD2A, PDPN, DUOX2	8.77E-4	4.516108108108108
GO:0005923	bicellular tight junction	3,38	CLDN4, POF1B, MYH14, MAPK15	0.001	1.7500418930888375
GO:0001533	cornified envelope	2,54	TGM1, PKP1, SPRR1B	0.001	3.6303119840097335
GO:0009925	basal plasma membrane	2,54	CLDN4, TACSTD2, BEST1	0.002	3.26823613086771

GO:0030175	filopodium	2,54	ACTA2, FARP1, PDPN	0.003	7.950894556528359
GO:0016324	apical plasma membrane	5,084	CLDN4, VANGL2, ABCC2, PDPN, DUOX2, PROM2	0.007	4.052917532917533
GO:0005794	Golgi apparatus	9,32	GASK1B, GALNT5, RAB25, TMEM30B, GALNT3, BDKRB2, MANSC1, NDRG2, B4GALNT3, MAPK15, FGFR2	0.008	21.16925675675676
GO:0043034	costamere	1,69	SYNM, FLNC	0.009	3.3577011956194114
GO:0005912	adherens junction	3,38	SYNM, POF1B, DLG5, PKP1	0.010	5.819726943438284

Table S4D. KEGG terms for the dataset of differentially expressed genes comparing HSC-3P with HSC-3R cell lines: terms for HSC-3R downregulated genes.

KEGGID	KEGG Term	% Associated Genes	Associated Genes	Term p-value corrected with Benjamini-Hochberg	Fold Enrichment
hsa04820	Cytoskeleton in muscle cells	5,08	COL4A1, LAMA1, TNNT3, MYH14, FLNC, ANKRD2	0.012	4.233716475095785
hsa04310	Wnt signaling pathway	4,23	VANGL2, CCND2, SFRP5, APCDD1L, FZD10	0.020	4.704129416773095
hsa05200	Pathways in cancer	6,77	CCND2, COL4A1, LAMA1, BDKRB2, PDGFA, BDKRB1, FZD10, FGFR2	0.038	2.4570912375790424
hsa00480	Glutathione metabolism	2,54	GPX2, GGT6, ANPEP	0.047	8.46743295019157
hsa04810	Regulation of actin cytoskeleton	4,23	BDKRB2, PDGFA, MYH14, BDKRB1, FGFR2	0.048	3.5587761674718195
hsa04390	Hippo signaling pathway	3,389	SOX2, CCND2, DLG5, FZD10	0.067	4.170794998820477
hsa04512	ECM-receptor interaction	2,54	COL4A1, LAMA1, NPNT	0.099	5.518102372034956
