# Supplementary material for: Generation and Characterization of Cisplatin-Resistant Oral Squamous Cell Carcinoma Cells Displaying an Epithelial–Mesenchymal Transition Signature
Source: Cells. 2025 Aug 24;14(17):1311. doi: 10.3390/cells14171311 (PMC12427644; doi:10.3390/cells14171311)
Supplement: Supplementary file 1 [file cells-14-01311-s001.zip › Table S5.pdf]

Table S5A. Gene ontology (GO) terms for SCC-9R upregulated genes.

GOID (Biological Process)	GO Term (Biological Process)	% Associated Genes	Associated Genes	Term p-value corrected with Benjamini-Hochberg	Fold Enrichment
GO:0030198	extracellular matrix organization	6,37	PDGFRA, COL18A1, OLFML2B, FOXF1, MMP2, ADAMTS10, COL1A1, ADAMTS15, ADAMTS5, COL3A1, ADAMTS2, COL1A2, COL5A1, ABI3BP, MMP17, COL5A2, PTX3, ADAMTS9, ADAMTS7	5.60E-11	7.685524535727463
GO:0030199	collagen fibril organization	4,02	COL1A1, COL18A1, FOXC2, COL3A1, TGFB2, ADAMTS2, COL1A2, COL5A1, LUM, COL12A1, COL5A2, ADAMTS7	1.81E-9	12.919148936170213
GO:0001525	angiogenesis	6,04	PDGFRB, COL18A1, ROBO4, NRP2, EDN2, CXCL8, PLXND1, MCAM, MMP2, NPR3, SERPINE1, PTPRJ, PRKCA, EREG, ANPEP, HMOX1, SCG2, FGFR1	1.79E-7	4.901233545823329
GO:0001649	osteoblast differentiation	4,36	LEF1, IGF2, TWIST1, FGF2, GLIS1, RUNX2, MRC2, COL1A1, GJA1, VCAN, CREB3L1, ITGA11, CCN4	3.56E-7	6.997872340425531
GO:0007155	cell adhesion	8,05	COL18A1, RIPOR2, NRP2, PCDHGB5, SEMA4D, PCDH9, MCAM, ITGB3, COL12A1, AMIGO2, PRKCA, COL1A1, VCAN, CXCL12, COL5A1,	1.55E-6	3.2485287460389314

			ADAM12, ITGA11, SPOCK1, CCN4, COL6A3, SVEP1, FERMT2, CNTNAP5, EPHA3		
GO:0001501	skeletal system development	4,02	HES7, COL1A1, COL18A1, TGFB2, VCAN, COL1A2, NPR3, COL5A2, TBX3, FGFR1, FBN1, CMKLR1	2.60E-6	6.459574468085107
GO:0030335	positive regulation of cell migration	5,70	PDGFRB, PDGFRA, SEMA4D, FOXF1, MCAM, MMP2, LEF1, TWIST1, PRKCA, COL1A1, PREX1, CXCL12, CEACAM6, PDGFC, MYADM, HAS2, FERMT2	3.91E-6	4.145081177255541
GO:0051897	positive regulation of phosphatidylinositol 3-kinase/protein kinase B signal transduction	4,69	PDGFRB, PDGFRA, TGFB2, SEMA4D, AKR1C3, IGF2, PTPRJ, MEIS3, FGF2, DCN, PDGFC, HPSE, FERMT2, FGFR1	3.96E-6	5.10261524822695
GO:0010595	positive regulation of endothelial cell migration	3,02	FOXC2, NRP2, SPARC, ADGRA2, ITGB3, ATOH8, WNT5A, PRKCA, FGF2	4.29E-6	9.542553191489361
GO:0042060	wound healing	3,02	PDGFRA, COL3A1, TGFB2, ITGB3, TPM1, WNT5A, NRG1, FGF2, EREG	1.51E-5	8.074468085106384
GO:0009887	animal organ morphogenesis	3,69	COL18A1, FOXF1, FHL1, PDGFC, IGF2, MEIS3, NKX6-1, FGF2, DCN, TBX3, EREG	2.25E-5	5.744522070498571
GO:0071230	cellular response to amino acid stimulus	2,68	COL1A1, PDGFRA, COL3A1, ZEB1, COL1A2, MMP2, PDGFC, COL5A2	3.42E-5	8.747340425531915

GO:0008284	positive regulation of cell population proliferation	7,04	PDGFRB, PDGFRA, PTGFR, TGFB2, EDN2, AKR1C3, IGF2, NRG1, MEIS3, FGF2, TBX3, EREG, PLAC8, AR, CEACAM6, PDGFC, HAS2, S1PR3, IL7R, CRLF1, FGFR1	4.16E-5	2.904255319148936
GO:0006935	~chemotaxis	3,35	SPN, RIPOR2, C5, CXCL12, CXCL8, ENPP2, FPR1, CXCL1, FGF2, CMKLR1	5.95E-5	5.783365570599614
GO:0010977	negative regulation of neuron projection development	2,68	DPYSL3, PTPRO, PMP22, SPOCK1, VIM, DKK1, PTPRG, ITM2C	6.72E-5	7.884926580761163
GO:0007517	muscle organ development	3,02	RIPOR2, TAGLN, SGCB, FHL1, ITGA11, TWIST1, COL6A3, DPF3, ETV1	8.54E-5	6.361702127659574
GO:0048661	positive regulation of smooth muscle cell proliferation	2,34	PDGFRB, ITGB3, CCN4, HMOX1, FGF2, TLR4, EREG	1.150E-4	9.071315996847911
GO:0008285	negative regulation of cell population proliferation	6,04	COL18A1, BCHE, TGFB2, CXCL8, ZBTB16, PTPRJ, CXCL1, P3H3, SMAD6, EREG, AR, ABI3BP, PMP22, ZNF503, SLIT3, TP53I11, TNS2, TP73	1.75E-4	2.902343366996764
GO:0010467	gene expression	3,35	SGCB, GRB10, ENPP1, SVEP1, IL7R, ROS1, TLR4, RUNX2, FGFR1, FBN1	1.92E-4	4.96303002867059
GO:0014911	positive regulation of smooth muscle cell migration	1,67	PDGFRB, ITGB3, CCN4, HAS2, TLR4	1.99E-4	16.66160081053698

GO:0009653	anatomical structure morphogenesis	3,35	FOXC2, MCAM, ATOH8, CLTCL1, FOXL2, S1PR3, FOXL1, SMAD6, EREG, FBN1	2.63E-4	4.760457374439137
GO:1900026	positive regulation of substrate adhesion-dependent cell spreading	2,01	PREX1, ITGB3, MYADM, HAS2, FERMT2, LIMS2	4.80E-4	9.127659574468085
GO:0001938	positive regulation of endothelial cell proliferation	2,34	NRP2, CXCL12, ITGB3, WNT5A, PRKCA, SCG2, FGF2	5.62E-4	6.803486997635934
GO:0043931	ossification involved in bone maturation	1,34	SEMA4D, ZBTB16, FAT4, ADAMTS7	7.35E-4	21.53191489361702
GO:0007229	integrin-mediated signaling pathway	2,68	COL3A1, ITGA10, ITGB3, ITGA11, ADAM12, FERMT2, ADAMTS10, LIMS2	0.001	5.089361702127659
GO:0060325	face morphogenesis	1,67	COL1A1, PDGFRA, MMP2, LEF1, DKK1	0.001	10.934175531914894
GO:0030324	lung development	2,34	PDGFRA, COL3A1, ADAMTS2, FOXF1, WNT5A, FGF2, SIM2	0.001	5.973793461338869
GO:0006954	inflammatory response	5,36	GGT5, PTGFR, CXCL8, PTGER2, WNT5A, FPR1, CXCL1, CXCL3, C5, DPEP1, CHST1, PTX3, S1PR3, SCG2, TLR4, CMKLR1	0.001	2.6532217404456993
GO:0048008	platelet-derived growth factor receptor signaling pathway	1,67	PDGFRB, PDGFRA, ITGB3, PDGFC, PTPRJ	0.001	10.602836879432624
GO:0030593	neutrophil chemotaxis	2,34	PREX1, TGFB2, EDN2, CXCL8, DPEP1, CXCL1, CXCL3	0.001	5.901820046142015
GO:0010718	positive regulation of epithelial to mesenchymal transition	2,01	COL1A1, TGFB2, TBX20, LEF1, TWIST1, FERMT2	0.001	7.497720364741641

GO:0071345	cellular response to cytokine stimulus	1,67	PID1, FOXF1, DPYSL3, LEF1, NKX6-1	0.001	10.290988735919901
GO:0000122	negative regulation of transcription by RNA polymerase II	9,06	FOXC2, LEF1, TWIST1, GLIS1, ZNF608, CREB3L1, TBX20, ZNF469, TBL1X, NKX6-1, MSC, SAMD11, HES7, SEMA4D, FOXF1, ZBTB16, IGF2, FOXL2, DKK1, TBX3, AR, ZEB2, ZEB1, BIN1, ZNF239, SIM2, FGFR1	0.001	1.9620202823622988
GO:0001503	ossification	2,34	COL1A1, FOXC2, SLC26A2, COL5A2, IGF2, TWIST1, RUNX2	0.001	5.695942602671945
GO:0001764	neuron migration	2,68	COL3A1, GJA1, CXCL12, SPOCK1, TWIST1, DISC1, FGFR1, ADGRL3	0.001	4.744320230796971
GO:0043542	endothelial cell migration	1,67	ZEB2, EDN2, PLXND1, ADGRA2, SCG2	0.001	9.719267139479905
GO:0003180	aortic valve morphogenesis	1,67	ADAMTS5, TBX20, TWIST1, SLIT3, SMAD6	0.001	9.456584243818288
GO:0071277	cellular response to calcium ion	2,34	TRPM2, SYT11, CPNE7, WNT5A, AKR1C3, DPEP1, KCNH1	0.001	5.382978723404255
GO:0043588	skin development	1,67	COL1A1, COL3A1, ADAMTS2, COL5A1, COL5A2	0.002	8.97163120567376
GO:0003203	endocardial cushion morphogenesis	1,34	ADAMTS5, TGFB2, TBX20, TWIST1	0.002	14.732362821948488
GO:0045766	positive regulation of angiogenesis	3,02	CXCL8, ITGB3, WNT5A, ADAM12, SERPINE1, HMOX1, TWIST1, PRKCA, FGF2	0.002	3.8638558934864897
GO:0010628	positive regulation of gene expression	5,70	PTGFR, PID1, CXCL8, ITGB3, LEF1, WNT5A, TWIST1, FGF2,	0.002	2.33720687204782

			DKK1, ACTG2, RUNX2, AR, GJA1, VIM, IL7R, TLR4, RBMS3		
GO:2000648	positive regulation of stem cell proliferation	1,67	GJA1, FGF2, RUNX2, TBX3, FGFR1	0.002	8.33080040526849
GO:0007411	axon guidance	3,35	SDK1, ROBO4, NRP2, CXCL12, SEMA4D, WNT5A, PTPRO, ETV1, SLIT3, EPHA3	0.003	3.3643617021276597
GO:0061028	establishment of endothelial barrier	1,34	ROBO4, PDE4D, MYADM, HPSE	0.003	13.329280648429584
GO:0001756	somitogenesis	1,67	HES7, FOXC2, FOXF1, WNT5A, LEF1	0.003	8.137060860959922
GO:0120162	positive regulation of cold-induced thermogenesis	2,34	PLAC8, GJA1, ACSL1, PDGFC, DIO2, GRB10, CMKLR1	0.003	4.850010532968191
GO:0050919	negative chemotaxis	1,67	NRP2, SEMA4D, ITGB3, SLIT3, NRG1	0.003	7.952127659574469
GO:0045892	negative regulation of DNA-templated transcription	6,04	ZBTB16, LEF1, WNT5A, TWIST1, NRG1, FOXL2, FOXN3, TBX3, RUNX2, EREG, ZEB2, ZEB1, CREB3L1, TBX20, ATOH8, ANKRD1, ZNF503, SAMD11	0.003	2.198284504845717
GO:0030154	cell differentiation	6,71	RIPOR2, FOXC2, FHL1, ILDR2, ETV1, NRG1, FOXL2, FOXL1, SMAD6, FGF2, GLIS1, ZEB1, DMBT1, ANPEP, ATOH8, PMP22, NKX6-1, ROS1, SIM2, FGFR1	0.004	2.0642691269691835

GO:0036120	cellular response to platelet-derived growth factor stimulus	1,34	PDGFRB, ITGB3, HAS2, TLR4	0.004	12.170212765957446
GO:0046627	negative regulation of insulin receptor signaling pathway	1,67	PID1, GRB10, ENPP1, PTPRJ, TNS2	0.004	7.6063829787234045
GO:0071260	cellular response to mechanical stimulus	2,01	COL1A1, RIPOR2, ITGB3, ANKRD1, FGF2, TLR4	0.004	5.452887537993921
GO:0007160	cell-matrix adhesion	2,34	COL3A1, ITGA10, ITGB3, ITGA11, SVEP1, HPSE, FERMT2	0.004	4.453191489361702
GO:0010629	negative regulation of gene expression	4,02	TGFB2, GJA1, CXCL8, CREB3L1, STC2, ATOH8, MYADM, ZNF503, SLIT3, ROS1, FGF2, RBMS3	0.005	2.70885380919698
GO:0051781	positive regulation of cell division	1,67	TGFB2, PDGFC, IGF2, FGF2, EREG	0.005	7.140686061658706
GO:0001568	blood vessel development	1,67	COL1A1, COL1A2, COL5A1, FOXF1, TBX3	0.005	7.140686061658706
GO:0003148	outflow tract septum morphogenesis	1,34	TGFB2, NRP2, TBX20, SMAD6	0.005	11.19659574468085
GO:0007156	homophilic cell adhesion via plasma membrane adhesion molecules	3,02	SDK1, ROBO4, PCDHGB5, PCDH9, CADM2, CEACAM6, AMIGO2, FAT4, HMCN1	0.005	3.3679599499374215
GO:0018108	peptidyl-tyrosine phosphorylation	1,67	PDGFRB, PDGFRA, EFEMP1, EPHA3, FGFR1	0.005	6.997872340425532
GO:0070374	positive regulation of ERK1 and ERK2 cascade	3,35	PDGFRB, PDGFRA, ITGB3, PDGFC, PRKCA, NRG1, TNFAIP8L3, FGF2, TLR4, FERMT2	0.005	3.0692422545726017

GO:0007162	negative regulation of cell adhesion	1,67	SPN, RIPOR2, PLXND1, SEMA4D, MMP2	0.005	6.860659157279933
GO:0010951	negative regulation of endopeptidase activity	1,67	SERPINE1, SPOCK1, SERPINA6, TIMP4, SERPINA5	0.005	6.860659157279933
GO:0048565	digestive tract development	1,34	COL3A1, FOXF1, PDGFC, FAT4	0.006	10.367218282111898
GO:0006198	cAMP catabolic process	1,00	PDE4D, PDE4A, PDE7B	0.006	23.326241134751772
GO:0007520	myoblast fusion	1,34	ADAMTS15, ADAMTS5, ADAM12, KCNH1	0.007	9.996960486322187
GO:0045944	positive regulation of transcription by RNA polymerase II	1,00	FOXC2, PID1, LEF1, TWIST1, FGF2, GLIS1, PLAC8, HSF2, CREB3L1, TBX20, ATOH8, TBL1X, NKX6-1, LUM, FOXF1, ZBTB16, WNT5A, IGF2, ETV1, FOXL2, MEIS3, TBX3, RUNX2, DCN, AR, TOX2, ZEB2, ZEB1, TLR4, TP73	0.007	1.6714663233500475
GO:0048146	positive regulation of fibroblast proliferation	1,67	PDGFRA, ITGB3, PDGFC, WNT5A, EREG	0.007	6.479511426319937
GO:0042733	embryonic digit morphogenesis	1,67	GJA1, ZBTB16, WNT5A, TWIST1, TBX3	0.007	6.479511426319937
GO:0030308	negative regulation of cell growth	2,34	TGFB2, GJA1, GNG4, FHL1, ENPP1, PTPRJ, SLIT3	0.007	4.08209219858156
GO:0001822	kidney development	2,34	TGFB2, PLXND1, WNT5A, MMP17, HAS2, TNS2, TP73	0.007	4.08209219858156
GO:0043406	positive regulation of MAP kinase activity	1,67	PDGFRB, PDGFC, FGF2, TLR4, FGFR1	0.007	6.361702127659575
GO:0032964	collagen biosynthetic process	1,00	COL1A1, COL5A1, P3H3	0.008	20.993617021276595

GO:0048048	embryonic eye morphogenesis	1,00	EFEMP1, FOXL2, FBN1	0.008	20.993617021276595
GO:0002062	chondrocyte differentiation	1,67	COL3A1, SLC26A2, RUNX2, FGFR1, ADAMTS7	0.009	6.032648569332356
GO:0036302	atrioventricular canal development	1,00	TBX20, HAS2, TBX3	0.010	19.085106382978722
GO:0001569	branching involved in blood vessel morphogenesis	1,34	FOXC2, PLXND1, TBX20, LEF1	0.010	8.747340425531915
GO:0045785	positive regulation of cell adhesion	1,67	CXCL12, TPM1, PTPRJ, PRKCA, NRG1	0.010	5.831560283687944
GO:0045597	positive regulation of cell differentiation	1,67	AR, LEF1, CCN4, DPF3, FGFR1	0.010	5.831560283687944
GO:1902895	positive regulation of miRNA transcription	1,67	AR, TGFB2, ATOH8, SMAD6, FGF2	0.010	5.831560283687944
GO:0007507	heart development	3,02	FOXC2, COL3A1, TGFB2, GJA1, FOXF1, MMP2, PTPRJ, FOXL1, FBN1	0.011	2.9707948615014046
GO:0060045	positive regulation of cardiac muscle cell proliferation	1,34	TBX20, NRG1, FGF2, FGFR1	0.011	8.4822695035461
GO:0048846	axon extension involved in axon guidance	1,00	NRP2, WNT5A, SLIT3	0.012	17.49468085106383
GO:0055003	cardiac myofibril assembly	1,00	PDGFRB, PDGFRA, ADPRHL1	0.012	17.49468085106383
GO:0006508	proteolysis	5,36	GGT5, HTRA3, MMP2, HTRA1, ADAMTS10, ADAMTS15, ADAMTS5, ADAMTS2, ANPEP, PAPPA, MMP17, ADAM12,	0.013	2.0210461632997925

			DPEP1, TBL1X, ADAMTS9, ADAMTS7		
GO:0001701	in utero embryonic development	3,02	PDGFRA, AR, COL3A1, GJA1, FOXF1, IGF2, TWIST1, TBX3, FGFR1	0.014	2.849812265331664
GO:0010572	positive regulation of platelet activation	1,00	SVEP1, PTPRJ, TLR4	0.014	16.148936170212764
GO:0045765	regulation of angiogenesis	1,34	PLXND1, ADGRA2, HMOX1, FGF2	0.014	7.775413711583924
GO:0060021	roof of mouth development	1,67	PDGFRA, CDK20, TWIST1, MSC, TBX3	0.014	5.301418439716312
GO:0001657	ureteric bud development	1,34	FOXC2, SMAD6, CRLF1, FGFR1	0.015	7.5652673950546285
GO:0062009	secondary palate development	1,00	TGFB2, WNT5A, LEF1	0.016	14.995440729483281
GO:0050850	positive regulation of calcium-mediated signaling	1,34	PDGFRB, PDGFRA, EDN2, PTPRJ	0.016	7.366181410974244
GO:0071560	cellular response to transforming growth factor beta stimulus	1,67	COL1A1, ZEB1, WNT5A, ANKRD1, FBN1	0.016	5.070921985815604
GO:0070098	chemokine-mediated signaling pathway	1,67	CXCL12, CXCL8, CXCL1, CXCL3, CMKLR1	0.017	4.998480243161095
GO:0043410	positive regulation of MAPK cascade	2,68	AR, IGFBP4, WNT5A, IGF2, PTPRJ, FGF2, FGFR1, TP73	0.018	2.977818017202354
GO:0032963	collagen metabolic process	1,00	COL1A2, P3H3, TNS2	0.018	13.995744680851065
GO:0050930	induction of positive chemotaxis	1,00	CXCL12, CXCL8, SCG2	0.018	13.995744680851065

GO:0030326	embryonic limb morphogenesis	1,34	TGFB2, LEF1, DKK1, FGFR1	0.019	6.997872340425532
GO:0071222	cellular response to lipopolysaccharide	2,68	CXCL8, WNT5A, SERPINE1, ANKRD1, CXCL1, VIM, CXCL3, TLR4	0.019	2.9310460064609556
GO:0030574	collagen catabolic process	1,34	MRC2, ADAMTS2, MMP2, MMP17	0.020	6.827192527244422
GO:0033627	cell adhesion mediated by integrin	1,34	ITGA10, ITGB3, ITGA11, FBN1	0.020	6.827192527244422
GO:0046325	negative regulation of glucose import	1,00	PID1, GRB10, ENPP1	0.021	13.121010638297873
GO:0033630	positive regulation of cell adhesion mediated by integrin	1,00	FOXC2, TGFB2, ITGB3	0.021	13.121010638297873
GO:0030514	negative regulation of BMP signaling pathway	1,67	HTRA3, WNT5A, HTRA1, SMAD6, DKK1	0.021	4.728292121909144
GO:0007169	cell surface receptor protein tyrosine kinase signaling pathway	2,01	PDGFRB, PDGFRA, NRG1, ROS1, PTPRG, FGFR1	0.021	3.7826336975273143
GO:0019933	cAMP-mediated signaling	1,34	PDE4D, PDE4A, PDE6A, PDE7B	0.021	6.664640324214792
GO:0060070	canonical Wnt signaling pathway	2,01	ADGRA2, WNT5A, LEF1, DISC1, FGF2, DKK1	0.022	3.7488601823708203
GO:0032722	positive regulation of chemokine production	1,34	C5, WNT5A, HMOX1, TLR4	0.023	6.509648688767936
GO:0001658	branching involved in ureteric bud morphogenesis	1,34	HS3ST3B1, HS3ST3A1, FAT4, FGF2	0.024	6.361702127659575
GO:0010812	negative regulation of cell-substrate adhesion	1,00	COL1A1, PTPRO, SPOCK1	0.026	11.663120567375886

GO:0060065	uterus development	1,00	TGFB2, WNT5A, FOXL2	0.026	11.663120567375886
GO:0071347	cellular response to interleukin-1	1,67	CXCL8, MMP2, ANKRD1, HAS2, ADAMTS7	0.027	4.373670212765958
GO:0002246	wound healing involved in inflammatory response	6,71	HMOX1, TLR4	0.028	69.97872340425532
GO:1903225	negative regulation of endodermal cell differentiation	6,71	COL5A1, COL5A2	0.028	69.97872340425532
GO:0060599	lateral sprouting involved in mammary gland duct morphogenesis	6,71	AR, WNT5A	0.028	69.97872340425532
GO:0120163	negative regulation of cold-induced thermogenesis	1,34	ADAMTS5, FOXC2, NPR3, TLR4	0.030	5.831560283687943
GO:0007165	signal transduction	9,06	OLFML2B, CXCL8, HHIP, NPR3, FPR1, CXCL1, FGF2, SPN, SYDE1, GJA1, CHN1, CCN4, PDE4A, PDE6A, PDGFRB, KLRC2, IGFBP4, PDE4D, P2RX6, AR, CXCL12, CEACAM6, CDC42EP3, TNFSF9, PDE7B, IL7R, FBN1	0.031	1.5237302676733013
GO:0007219	Notch signaling pathway	2,01	HES7, FOXC2, SORBS2, S1PR3, FAT4, TIMP4	0.031	3.413596263622211
GO:0050731	positive regulation of peptidyl-tyrosine phosphorylation	1,67	SEMA4D, ITGB3, ENPP2, IGF2, PTPRJ	0.031	4.165400202634245

GO:2000406	positive regulation of T cell migration	1,00	SPN, CXCL12, ITGB3	0.032	10.496808510638298
GO:0072089	stem cell proliferation	1,34	FGF2, RUNX2, TBX3, FGFR1	0.032	5.712548849326964
GO:0006357	regulation of transcription by RNA polymerase II	1,10	FOXC2, LEF1, TWIST1, GLIS1, ZNF608, HSF2, CREB3L1, TBX20, ANKRD1, HMOX1, DPF3, TBL1X, NKX6-1, MSC, HES7, FOXF1, ZBTB16, TSC22D3, ZNF98, ETV1, FOXL2, FOXN3, FOXL1, SMAD6, TBX3, RUNX2, RFX8, TOX2, ZEB2, ZEB1, ZNF239, SIM2, TP73	0.033	1.4379189740600407
GO:0030500	~regulation of bone mineralization	1,00	GJA1, TWIST1, ENPP1	0.035	9.99696048632219
GO:0003184	pulmonary valve morphogenesis	1,00	ADAMTS5, TGFB2, SMAD6	0.035	9.99696048632219
GO:0045666	positive regulation of neuron differentiation	1,67	CXCL12, ZEB1, ZC4H2, NKX6-1, FGFR1	0.035	4.021765712888237
GO:0030879	mammary gland development	1,00	LEF1, NRG1, TBX3	0.038	9.542553191489361
GO:0030318	melanocyte differentiation	1,00	LRMDA, ENPP1, ADAMTS9	0.038	9.542553191489361
GO:0040037	negative regulation of fibroblast growth factor receptor signaling pathway	1,00	CREB3L1, WNT5A, FGF2	0.038	9.542553191489361
GO:0060326	cell chemotaxis	1,67	PDGFRB, PDGFRA, C5, CXCL12, LEF1	0.039	3.8877068557919623
GO:0001837	epithelial to mesenchymal transition	1,34	TGFB2, WNT5A, LEF1, FGFR1	0.041	5.183609141055949

GO:0032331	negative regulation of chondrocyte differentiation	1,00	EFEMP1, CCN4, ADAMTS7	0.041	9.127659574468085
GO:0070236	negative regulation of activation-induced cell death of T cells	6,71	GPAM, TSC22D3	0.042	46.652482269503544
GO:0010966	regulation of phosphate transport	6,71	ROS1, FGFR1	0.042	46.652482269503544
GO:0038091	positive regulation of cell proliferation by VEGF-activated platelet derived growth factor receptor signaling pathway	6,71	PDGFRB, PDGFRA	0.042	46.652482269503544
GO:0072277	metanephric glomerular capillary formation	6,71	PDGFRB, PDGFRA	0.042	46.652482269503544
GO:0045726	positive regulation of integrin biosynthetic process	6,71	AR, TGFB2	0.042	46.652482269503544
GO:0033674	positive regulation of kinase activity	1,34	PDGFRB, PDGFRA, ROS1, FGFR1	0.043	5.089361702127659
GO:0050680	negative regulation of epithelial cell proliferation	1,34	AR, TGFB2, WNT5A, EREG	0.043	5.089361702127659
GO:0071356	cellular response to tumor necrosis factor	2,01	COL1A1, CXCL8, PID1, ANKRD1, HAS2, ADAMTS7	0.045	3.08729662077597
GO:0008360	regulation of cell shape	2,01	PLXND1, SEMA4D, TPM1, CDC42EP3, RHOU, FERMT2	0.047	3.0425531914893615

GO:0032956	regulation of actin cytoskeleton organization	1,67	PDGFRB, PDGFRA, TRPM2, ITGB3, EPHA3	0.048	3.6447251773049647
GO:0042327	positive regulation of phosphorylation	1,00	AR, GRB10, EREG	0.048	8.397446808510638
GO:0002053	positive regulation of mesenchymal cell proliferation	1,00	FOXF1, WNT5A, FGFR1	0.048	8.397446808510638
GO:0060038	cardiac muscle cell proliferation	1,00	FOXC2, TGFB2, FGFR1	0.048	8.397446808510638
GO:0008543	fibroblast growth factor receptor signaling pathway	1,34	WNT5A, FAT4, FGF2, FGFR1	0.049	4.826118855465884
GO:0045599	negative regulation of fat cell differentiation	1,34	WNT5A, CCN4, ENPP1, FERMT2	0.049	4.826118855465884

Table S5B. Gene Ontology (GO) of molecular function: terms for SCC-9R upregulated genes.

GOID (Molecular Function)	GO Term (Molecular Function)	% Associated Genes	Associated Genes	Term p-value corrected with Benjamini-Hochberg	Fold Enrichment
GO:0005178	integrin binding	5,36	ITGB3, IGF2, PRKCA, NRG1, FGF2, ADAMTS5, COL3A1, CXCL12, COL5A1, ITGA10, ITGA11, CCN4, S1PR3, SVEP1, FERMT2, FBN1	1.86E-8	6.666321332988
GO:0048407	platelet-derived growth factor binding	2,01	PDGFRB, COL1A1, PDGFRA, COL3A1, COL1A2, COL5A1	4.92E-7	33.74825174825175
GO:0008237	metallopeptidase activity	3,02	ADAMTS5, ADAMTS2, ANPEP, MMP2, PAPPA, ADAM12, ADAMTS9, ADAMTS10, ADAMTS7	3.46E-6	9.797879539815025
GO:0050840	extracellular matrix binding	2,34	ADAMTS15, ADAMTS5, SPARC, OLFML2B, ITGB3, SPOCK1, DCN	7.78E-6	14.317440135621954
GO:0030020	extracellular matrix structural constituent conferring tensile strength	2,68	COL1A1, COL18A1, COL3A1, COL1A2, COL5A1, COL12A1, COL5A2, COL6A3	1.38E-5	9.999481999482
GO:0008201	heparin binding	4,36	NRP2, CXCL8, FGF2, SERPINA5, COL1A1, ADAMTS15, ADAMTS5, COL5A1, ABI3BP, CCN4, SLIT3, FGFR1, FBN1	2.41E-5	4.667311411992263
GO:0005161	platelet-derived growth factor receptor binding	1,67	PDGFRB, PDGFRA, ITGB3, PDGFC, PTPRJ	4.14E-5	24.10589410589411

GO:0004222	metalloendopeptidase activity	3,35	ADAMTS15, ADAMTS5, ADAMTS2, MMP2, PAPPA, MMP17, ADAM12, ADAMTS9, ADAMTS10, ADAMTS7	5.26E-5	5.869261173609
GO:0004115	3',5'-cyclic-AMP phosphodiesterase activity	1,67	PDE4D, PDE4A, ENPP1, PDE6A, PDE7B	2.75E-4	15.340114431023524
GO:0005201	extracellular matrix structural constituent	3,02	COL1A1, COL3A1, SPARC, EFEMP1, COL1A2, LUM, ABI3BP, HMCN1, FBN1	3.47E-4	5.192038730500269
GO:0005518	collagen binding	2,34	MRC2, SPARC, LUM, ABI3BP, ITGA10, ITGA11, SPOCK1	5.40E-4	6.8474713692105
GO:0008083	growth factor activity	3,35	TGFB2, CXCL12, EFEMP1, PDGFC, IGF2, CXCL1, NRG1, FGF2, DKK1, EREG	7.91E-4	4.090697181606273
GO:0045236	CXCR chemokine receptor binding	1,34	CXCL12, CXCL8, CXCL1, CXCL3	0.001	17.999067599067597
GO:0046872	metal ion binding	2,04	COL18A1, NRP2, SPO11, ITGB3, CPNE7, FHL1, ATP2A3, ACSM5, ZFYVE28, ADAMTS5, ZNF608, PDE4A, DPF3, ENPP1, ADAMTS9, TIMP4, TNS2, ADAMTS7, SAMD11, TNS1, PDGFRB, PDE4D, ZNF98, PRKCA, ZEB2, ZEB1, KCNMA1, ADAM12, DPEP1, ZNF239, RHOU, NPTXR, DGKI, MT1E, ZC4H2, PRUNE2, ADAMTS10, GLIS1, CHN1, HMOX1, ZNF503, ZNF469, LONRF3, PDE6A,	0.001	1.4641844641844641

			ATP8B3, ATP8B2, ZBTB16, SMAD6, SH3RF3, COL1A1, COL3A1, COL1A2, COL5A1, ITGA10, ZUP1, COL5A2, ITGA11, PDE7B, CDK15, LIMS2, TP73		
GO:0042802	identical protein binding	1,40	SMIM3, RIPOR2, FOXC2, NRP2, OLFML2B, HTRA3, ITGB3, HTRA1, GRIK2, FGF2, PTPRG, ADAMTS5, HSF2, DPYSL3, GRB10, HMOX1, HAS2, TBL1X, DISC1, TNS2, BCHE, SEMA4D, ZBTB16, MID1IP1, TPM1, SORBS2, SMAD6, DNM1, COL1A1, SDK1, COL1A2, CEACAM6, BIN1, SYT11, KCNMA1, GOPC, PTX3, VIM, TLR4, FGFR1, FBN1, TP73	0.001	1.6320398082056113
GO:0002020	protease binding	2,68	COL1A1, COL3A1, COL1A2, BIN1, ITGB3, SERPINE1, TIMP4, SERPINA5	0.001	4.576034135356169
GO:0005102	signaling receptor binding	4,69	PDGFRB, TGFB2, SEMA4D, IGFBP4, SERPINE1, CXCL1, NRG1, C1QL1, AR, GJA1, C5, CXCL12, TNFSF9, TLR4	0.003	2.560842951086854
GO:0047555	3',5'-cyclic-GMP phosphodiesterase activity	1,34	PDE4D, PDE4A, PDE6A, PDE7B	0.003	12.272091544818819
GO:0046332	SMAD binding	1,67	COL3A1, COL1A2, CREB3L1, COL5A2, FERMT2	0.005	7.030885780885781

GO:0003700	DNA-binding transcription factor activity	5,70	ZBTB16, LEF1, ETV1, FOXL2, FOXN3, FOXL1, RUNX2, AR, ZEB2, ZEB1, HSF2, TBX20, ATOH8, ZNF469, MSC, SIM2, TP73	0.006	2.1407473123891037
GO:0008009	chemokine activity	1,67	C5, CXCL12, CXCL8, CXCL1, CXCL3	0.006	6.617304264363088
GO:0005509	calcium ion binding	7,04	MEGF6, SPARC, PCDHGB5, PCDH9, CALB2, TRPM2, MEX3B, VCAN, PLCB4, EFEMP1, SYT11, ENPP2, SPOCK1, ENPP1, SVEP1, SLIT3, HMCN1, FAT4, MYL9, ADGRL3, FBN1	0.007	1.900035621215246
GO:0008146	sulfotransferase activity	1,34	HS3ST3B1, SULT1B1, HS3ST3A1, CHST1	0.014	7.7138861138861134
GO:0001228	DNA-binding transcription activator activity, RNA polymerase II-specific	5,03	FOXC2, FOXF1, ZBTB16, LEF1, ETV1, FOXL2, MEIS3, TBX3, GLIS1, RUNX2, AR, HSF2, CREB3L1, TBX20, TP73	0.015	2.053646151009234
GO:0048018	receptor ligand activity	1,67	SEMA4D, WNT5A, IGF2, NRG1, EREG	0.020	4.753274894119965
GO:0030021	extracellular matrix structural constituent conferring compression resistance	1,00	VCAN, LUM, DCN	0.022	12.655594405594407
GO:0008270	zinc ion binding	7,38	HHIP, MMP2, PRKCA, FBXO43, PTPRG, RTL3, ADAMTS15, TRIM9, AR, ADAMTS5, ADAMTS2, ZEB1, ANPEP,	0.025	1.6572802197802199

			PAPPA, MMP17, ENPP2, DPEP1, DPF3, ENPP1, ADAMTS9, MARCHF3, MT1E		
GO:0030297	transmembrane receptor protein tyrosine kinase activator activity	1,00	IGF2, NRG1, EREG	0.028	11.249417249417249
GO:0033872	[heparan sulfate]-glucosamine 3-sulfotransferase 3 activity	6,71	HS3ST3B1, HS3ST3A1	0.029	67.4965034965035
GO:0015179	L-amino acid transmembrane transporter activity	1,00	SLC43A1, SLC7A2, SLC38A4	0.034	10.124475524475525
GO:0005158	insulin receptor binding	1,00	GRB10, IGF2, ENPP1	0.041	9.204068658614114
GO:0001227	DNA-binding transcription repressor activity, RNA polymerase II-specific	3,69	ZEB2, ZEB1, CREB3L1, ZBTB16, LEF1, ZNF239, NKX6-1, MSC, GLIS1, TBX3, SAMD11	0.042	2.0566801619433197
GO:0038023	signaling receptor activity	2,68	MRC2, ROBO4, NRP2, SEMA4D, ANPEP, TLR4, DCBLD1, CMKLR1	0.050	2.39987567987568

Table S5C. Gene Ontology (GO) of cellular component: terms for SCC-9R upregulated genes.

GOID (Cellular Component)	GO Term (Cellular Component)	% Associated Genes	Associated Genes	Term p-value corrected with Benjamini-Hochberg	Fold Enrichment
GO:0005615	extracellular space	2,34	COL18A1, SPARC, OLFML2B, CXCL8, COL12A1, SERPINE1, CXCL1, SERPINA6, CXCL3, FGF2, ACTG2, SERPINA5, SPN, ADAMTS5, EFEMP1, DMBT1, ANPEP, DPYSL3, ENPP2, CCN4, ENPP1, SVEP1, ADAMTS9, TIMP4, SRGN, EDN2, IGFBP4, MMP2, WNT5A, NRG1, DKK1, DCN, EREG, VCAN, CEACAM6, PAPPA, MMP17, DPEP1, COL6A3, SCG2, TLR4, GCNT1, HTRA1, KRT86, ADAMTS15, C5, FAM184A, ABI3BP, STC2, PDGFC, SPOCK1, HMOX1, SLIT3, BCHE, TGFB2, MGAM, SEMA4D, LUM, MCAM, IGF2, COL1A1, COL3A1, COL1A2, COL5A1, COL5A2, TNFSF9, PTX3, HPSE, CRLF1, FBN1	1.82E-12	2.4629402529496187
GO:0031012	extracellular matrix	7,718	OLFML2B, LUM, MMP2, WNT5A, ADAMTS10, COL1A1, ADAMTS15, ADAMTS5, ADAMTS2, VCAN, EFEMP1, DMBT1, MMP17, COL5A2, CCN4, COL6A3, PTX3, HPSE,	3.34E-12	6.85503710045662

			ADAMTS9, TLR4, TIMP4, ADAMTS7, FBN1		
GO:0062023	collagen-containing extracellular matrix	9,39	COL18A1, SPARC, COL12A1, SERPINE1, HTRA1, ADAMTS10, SERPINA5, ADAMTS15, ADAMTS5, ADAMTS2, EFEMP1, ABI3BP, HMCN1, ADAMTS9, TGFB2, LUM, MMP2, WNT5A, DCN, COL1A1, COL3A1, VCAN, CXCL12, COL1A2, COL5A1, COL5A2, COL6A3, FBN1	2.39E-11	4.9089779747515445
GO:0005576	extracellular region	2,18	COL18A1, NRP2, SPARC, CXCL8, HHIP, COL12A1, SERPINE1, CXCL1, SERPINA6, CXCL3, FGF2, SERPINA5, ADAMTS5, ADAMTS2, EFEMP1, DMBT1, CCN4, ENPP1, SVEP1, ADAMTS9, ADAMTS7, SRGN, EDN2, IGFBP4, MMP2, WNT5A, NRG1, DKK1, DCN, EREG, VCAN, PAPPA, ADAM12, SCG5, COL6A3, EPHA3, PTGFR, MEGF6, HTRA3, HTRA1, ADAMTS10, GLB1L2, PLAC8, C5, ABI3BP, PDGFC, BCHE, TGFB2, ERVMER34-1, LUM, MCAM, IGF2, ENOX1, COL1A1, COL3A1, CXCL12, COL1A2, COL5A1, COL5A2, PTX3, HPSE, IL7R, CRLF1, FBN1, FGFR1	1.30E-9	2.2129954424833578

GO:0005886	plasma membrane	4,02	CHIC2, SPARC, PLXND1, HHIP, CPNE7, SERPINE1, PREX1, GJA1, DMBT1, ADGRA2, ANPEP, ENPP2, GRB10, ENPP1, SVEP1, ROS1, TNS2, KCNH1, CMKLR1, PDGFRB, PDGFRA, WNT5A, DIO2, PRKCA, DKK1, EREG, AR, BIN1, ADAM12, MYADM, ELMO1, DPEP1, DGKI, EPHA3, PTGFR, SLC26A2, NPR3, HTRA1, FPR1, NKAIN2, TRPM2, PDGFC, SLC38A4, GGT5, MGAM, ATP8B3, ATP8B2, KLRC2, CADM2, MCAM, APCDD1L, SDK1, ITGA10, ITGA11, TNFSF9, FAT4, RGL1, IL7R, FERMT2, ITM2C, FGFR1, ROBO4, NRP2, PCDHGB5, CNRIP1, ITGB3, PTPRO, FHL1, PTPRJ, GRIK2, PTPRG, SPN, TMEM47, SGCB, PDE4A, FLVCR2, TNFAIP8L3, ABCC3, HS3ST3B1, PDE4D, MMP2, NRG1, KCNAB2, DNM1, PLCB4, CEACAM6, CDC42EP3, KCNMA1, MMP17, GAS1, RHOU, TLR4, PTGER2, SLC43A1, AMIGO2, SLC5A12, SLC7A2, P2RY8, C10ORF90, GNG4, HAS2, S1PR3, PDE6A,	1.02E-7	1.5349961784937385

			BCHE, ERVMER34-1, SEMA4D, PCDH9, SORBS2, DCBLD1, ENOX1, P2RX6, GPAM, SYT11, PMP22, GOPC, VIM, EVI2B, MPHOSPH8, LIMS2, ADGRL3		
GO:0005788	endoplasmic reticulum lumen	6,04	COL18A1, BCHE, IGFBP4, WNT5A, COL12A1, COL1A1, ADAMTS5, COL3A1, VCAN, COL1A2, COL5A1, STC2, PDGFC, COL5A2, COL6A3, SCG2, ADAMTS7, FBN1	1.92E-6	4.140047570805621
GO:0005581	collagen trimer	3,02	COL1A1, COL18A1, COL3A1, COL1A2, COL5A1, COL12A1, COL5A2, COL6A3, C1QL1	7.91E-5	6.437773972602739
GO:0016324	apical plasma membrane	5,36	PDGFRB, RIPOR2, MGAM, SLC26A2, PTPRQ, ITGB3, PDE4D, PTPRO, SLC43A1, SLC5A12, NRG1, SORBS2, GJA1, CEACAM6, KCNMA1, DPEP1	6.80E-4	2.791446708987638
GO:0098978	glutamatergic synapse	5,36	NRP2, PLXND1, ITGB3, PTPRO, WNT5A, NRG1, GRIK2, DNM1, DOCK10, P2RX6, PLCB4, BIN1, NPTXR, DISC1, DGKI, ADGRL3	0.001	2.6492897006595637
GO:0043235	receptor complex	3,69	PDGFRB, PDGFRA, P2RX6, KLRC2, PTPRQ, ITGB3, PTPRJ, ROS1, TLR4, CRLF1, FGFR1	0.001	3.4970624048706243
GO:0005604	basement membrane	2,34	SPN, COL18A1, SPARC, COL5A1, FAT4, HMCN1, FBN1	0.002	5.162018076542862
GO:0030424	axon	4,69	TGFB2, ROBO4, NRP2, PLXND1, CADM2, PTPRO, SDK1, SYT11,	0.002	2.635346070656092

			BIN1, HMCN1, VIM, DGKI, KCNH1, ADGRL3		
GO:0009897	external side of plasma membrane	4,69	PDGFRA, KLRC2, MCAM, ITGB3, SERPINA5, ENOX1, SPN, CXCL12, ANPEP, ITGA10, ITGA11, IL7R, TLR4, CRLF1	0.007	2.33433917680493
GO:0005925	focal adhesion	4,69	PDGFRB, MCAM, ITGB3, FHL1, SORBS2, MRC2, GJA1, ITGA11, RHOU, VIM, FERMT2, TNS2, LIMS2, TNS1	0.008	2.302141395055896
GO:0009986	cell surface	6,04	SPARC, ITGB3, HHIP, WNT5A, PTPRJ, SPN, ADAMTS15, DMBT1, CEACAM6, ADGRA2, PDGFC, ENPP1, ADAMTS9, ROS1, TLR4, FERMT2, ADAMTS7, TNS1	0.011	1.9597485456933759
GO:0031528	microvillus membrane	1,34	SLC26A2, ITGB3, DPEP1, SLC38A4	0.011	8.41539081385979
GO:0005587	collagen type IV trimer	1,00	COL1A1, COL1A2, COL5A2	0.013	16.507112750263435
GO:0031093	platelet alpha granule lumen	1,67	SRGN, TGFB2, SPARC, SERPINE1, IGF2	0.014	5.259619258662369
GO:0000785	chromatin	8,72	FOXC2, LEF1, TWIST1, HSF2, CREB3L1, TBX20, ATOH8, DPF3, NKX6-1, MSC, HES7, FOXF1, ETV1, FOXL2, FOXN3, FOXL1, MEIS3, SMAD6, TBX3, RUNX2, AR, RFX8, ZEB2, ZEB1, SIM2, TP73	0.019	1.6088247144143717
GO:0016020	membrane	2,91	ROBO4, NRP2, SPARC, PLXND1, PTPRQ, PTPRO, ATP2A3,	0.019	1.2328014078544602

			PTPRG, SERPINA5, SPN, MRC2, DOCK10, DMBT1, ADGRA2, CREB3L1, ENPP2, PDE4A, ENPP1, FLVCR2, SVEP1, ROS1, KCNH1, CMKLR1, ABCC3, PDGFRB, HS3ST3B1, PDGFRA, NUP210, ACSL1, PDE4D, TPM1, WNT5A, DIO2, NRG1, KCNAB2, DNM1, AR, VCAN, BIN1, CEACAM6, PAPPA, KCNMA1, ADAM12, CDC42EP3, ELMO1, GAS1, CHST1, NPTXR, TLR4, CNTNAP5, DGKI, SMIM3, SLC26A2, HS3ST3A1, PRUNE2, NPR3, GCNT1, AMIGO2, FPR1, ILDR2, TMTC1, P2RY8, TRPM2, CLTCL1, PDGFC, HMOX1, TP53I11, SLC38A4, MGAM, ATP8B3, SEMA4D, MCAM, C14ORF132, SHISAL1, SMAD6, DCBLD1, COL1A1, EVI2A, ITGA11, PMP22, GOPC, FAT4, HPSE, EVI2B, FBN1, FGFR1, ADGRL3		
GO:0002116	semaphorin receptor complex	1,00	NRP2, PLXND1, SEMA4D	0.022	12.623086220789684
GO:0045202	synapse	4,69	CADM2, ITGB3, SORBS2, KCNAB2, DNM1, CALB2, SDK1, VCAN, SYT11, DPYSL3, EEF1A2, PMP22, PDE7B, DGKI	0.025	1.9830326868303267

GO:0005584	collagen type I trimer	6,71	COL1A1, COL1A2	0.027	71.53082191780823
GO:0070062	extracellular exosome	1,44	COL18A1, ROBO4, SLC26A2, PCDHGB5, ITGB3, CPNE7, NPR3, COL12A1, PTPRO, SERPINE1, HTRA1, SLC5A12, PTPRJ, SERPINA6, HNMT, ACTG2, PTPRG, SERPINA5, DOCK10, SPN, C5, EFEMP1, DMBT1, GNG4, ANPEP, CLTCL1, PDGFC, HMCN1, NKX6-1, MGAM, LUM, WNT5A, AKR1C3, PRKCA, DNM1, CXCL12, COL1A2, KRT37, DPEP1, COL6A3, VIM, FAT4, ITM2C	0.031	1.3621901428103427
GO:0098688	parallel fiber to Purkinje cell synapse	1,00	CALB2, P2RX6, PLCB4	0.034	10.218688845401173
GO:0031258	lamellipodium membrane	1,00	PLXND1, ITGB3, FERMT2	0.040	9.330107206670638
GO:0035866	alphav-beta3 integrin-PKCalpha complex	6,71	ITGB3, PRKCA	0.041	47.687214611872136
GO:0031674	I band	1,00	BIN1, ANKRD1, FERMT2	0.043	8.941352739726026
GO:0030054	cell junction	2,34	PDGFRA, P2RX6, GJA1, DPEP1, FERMT2, SLC7A2, TP73	0.047	2.6776243498644785
GO:0043202	lysosomal lumen	1,67	PDGFRB, VCAN, LUM, HPSE, DCN	0.048	3.6495317305004193

Table S5D. KEGG terms for the dataset of differentially expressed genes comparing SCC-9R with SCC-9P cell lines: terms for SCC-9R upregulated genes.

KEGGID	KEGG Term	% Associated Genes	Associated Genes	Term p-value corrected with Benjamini-Hochberg	Fold Enrichment
hsa04820	Cytoskeleton in muscle cells	6,37	ITGB3, TPM1, FHL1, DCN, ACTG2, COL1A1, COL3A1, VCAN, COL1A2, COL5A1, SGCB, ITGA10, COL5A2, ITGA11, ANKRD1, COL6A3, VIM, MYL9, FBN1	9.83E-9	5.4027277406073075
hsa05205	Proteoglycans in cancer	5,03	TGFB2, LUM, ITGB3, MMP2, WNT5A, IGF2, TWIST1, PRKCA, FGF2, DCN, COL1A1, COL1A2, HPSE, TLR4, FGFR1	2.12E-6	4.850746268656716
hsa04151	PI3K-Akt signaling pathway	6,37	PDGFRB, PDGFRA, ITGB3, IGF2, PRKCA, FGF2, EREG, COL1A1, COL1A2, PPP2R2C, GNG4, ITGA10, CREB3L1, PDGFC, ITGA11, COL6A3, IL7R, TLR4, FGFR1	7.30E-6	3.4625216459140757
hsa05146	Amoebiasis	3,35	COL1A1, COL3A1, TGFB2, PLCB4, CXCL8, COL1A2, CXCL1, PRKCA, CXCL3, TLR4	2.28E-5	6.404868859585567
hsa04933	AGE-RAGE signaling pathway in diabetic complications	3,02	COL1A1, COL3A1, TGFB2, PLCB4, CXCL8, COL1A2, MMP2, SERPINE1, PRKCA	1.32E-4	5.8785281513225955
hsa05200	Pathways in cancer	6,71	PDGFRB, PDGFRA, TGFB2, CXCL8, PTGER2, HHIP, ZBTB16,	3.69E-4	2.4754277393520203

			MMP2, LEF1, WNT5A, IGF2, PRKCA, FGF2, AR, CXCL12, PLCB4, GNG4, HMOX1, IL7R, FGFR1		
hsa05215	Prostate cancer	2,68	PDGFRB, PDGFRA, AR, ZEB1, CREB3L1, PDGFC, LEF1, FGFR1	6.61E-4	5.385318306427048
hsa04510	Focal adhesion	3,69	PDGFRB, COL1A1, PDGFRA, COL1A2, ITGA10, ITGB3, ITGA11, PDGFC, COL6A3, PRKCA, MYL9	9.48E-4	3.5747371516800235
hsa04974	Protein digestion and absorption	2,68	COL1A1, COL18A1, COL3A1, COL1A2, COL5A1, COL12A1, COL5A2, COL6A3	9.97E-4	5.0262970859985785
hsa04928	Parathyroid hormone synthesis, secretion and action	2,68	PLCB4, CREB3L1, PDE4D, MMP17, PDE4A, PRKCA, RUNX2, FGFR1	0.001	4.589227774172615
hsa04072	Phospholipase D signaling pathway	3,02	PDGFRB, PDGFRA, PTGFR, PLCB4, CXCL8, PDGFC, PRKCA, DGKI, DNM1	0.001	3.984774115997195
hsa05412	Arrhythmogenic right ventricular cardiomyopathy	2,34	GJA1, SGCB, ITGA10, ITGB3, ITGA11, LEF1, ATP2A3	0.001	5.369663311350226
hsa04810	Regulation of actin cytoskeleton	3,69	PDGFRB, PDGFRA, C5, CXCL12, ITGA10, ITGB3, ITGA11, PDGFC, FGF2, MYL9, FGFR1	0.002	3.155094094743673
hsa04926	Relaxin signaling pathway	2,68	COL1A1, COL3A1, PLCB4, COL1A2, GNG4, CREB3L1, MMP2, PRKCA	0.003	4.059701492537314
hsa05410	Hypertrophic cardiomyopathy	2,34	TGFB2, SGCB, ITGA10, ITGB3, TPM1, ITGA11, ATP2A3	0.003	4.66455600783959

hsa04015	Rap1 signaling pathway	3,35	PDGFRB, PDGFRA, PLCB4, ITGB3, PDGFC, FPR1, PRKCA, SIPA1L2, FGF2, FGFR1	0.004	3.1117994931005346
hsa05414	Dilated cardiomyopathy	2,34	TGFB2, SGCB, ITGA10, ITGB3, TPM1, ITGA11, ATP2A3	0.004	4.398009950248756
hsa01521	EGFR tyrosine kinase inhibitor resistance	2,01	PDGFRB, PDGFRA, PDGFC, PRKCA, NRG1, FGF2	0.006	4.94776119402985
hsa05134	Legionellosis	1,67	CXCL8, EEF1A2, CXCL1, CXCL3, TLR4	0.009	5.890191897654583
hsa04512	ECM-receptor interaction	2,01	COL1A1, COL1A2, ITGA10, ITGB3, ITGA11, COL6A3	0.010	4.447425792386382
hsa04540	Gap junction	2,01	PDGFRB, PDGFRA, GJA1, PLCB4, PDGFC, PRKCA	0.012	4.302401038286827
hsa05323	Rheumatoid arthritis	2,01	TGFB2, CXCL12, CXCL8, CXCL1, CXCL3, TLR4	0.013	4.210860590663702
hsa04020	Calcium signaling pathway	3,35	PDGFRB, PDGFRA, P2RX6, PTGFR, PLCB4, PDGFC, ATP2A3, PRKCA, FGF2, FGFR1	0.014	2.597249970619344
hsa04310	Wnt signaling pathway	2,68	PLCB4, WNT5A, LEF1, APCDD1L, CCN4, PRKCA, TBL1X, DKK1	0.015	3.0331103105163835
hsa05142	Chagas disease	2,01	TGFB2, PLCB4, CXCL8, PPP2R2C, SERPINE1, TLR4	0.019	3.84292131575134
hsa05163	Human cytomegalovirus infection	3,02	PDGFRA, CXCL12, PLCB4, CXCL8, GNG4, CREB3L1, PTGER2, ITGB3, PRKCA	0.020	2.6271298375379737
hsa05218	Melanoma	1,67	PDGFRB, PDGFRA, PDGFC, FGF2, FGFR1	0.023	4.5185033735432425
hsa04062	Chemokine signaling pathway	2,68	PREX1, CXCL12, PLCB4, CXCL8, GNG4, ELMO1, CXCL1, CXCL3	0.026	2.73451395870389

hsa04014	Ras signaling pathway	3,02	PDGFRB, PDGFRA, GNG4, PDGFC, IGF2, PRKCA, RGL1, FGF2, FGFR1	0.027	2.4946695095948828
hsa05165	Human papillomavirus infection	3,69	PDGFRB, HES7, COL1A1, COL1A2, PPP2R2C, CREB3L1, ITGA10, ITGB3, ITGA11, WNT5A, COL6A3	0.028	2.179194119492627
hsa05415	Diabetic cardiomyopathy	2,68	COL1A1, COL3A1, TGFB2, PLCB4, COL1A2, MMP2, ATP2A3, PRKCA	0.034	2.574444848926101
hsa05225	Hepatocellular carcinoma	2,34	TGFB2, WNT5A, LEF1, IGF2, HMOX1, DPF3, PRKCA	0.043	2.716417910447761
hsa04961	Endocrine and other factor-regulated calcium reabsorption	1,34	PLCB4, CLTCL1, PRKCA, DNM1	0.045	4.978879188960856
hsa05206	MicroRNAs in cancer	3,35	PDGFRB, PDGFRA, TGFB2, ZEB2, ZEB1, ITGB3, TPM1, HMOX1, PRKCA, VIM	0.045	2.1144278606965172
hsa05032	Morphine addiction	1,67	GNG4, PDE4D, PDE4A, PRKCA, PDE7B	0.048	3.624733475479744
