# Supplementary material for: Generation and Characterization of Cisplatin-Resistant Oral Squamous Cell Carcinoma Cells Displaying an Epithelial–Mesenchymal Transition Signature
Source: Cells. 2025 Aug 24;14(17):1311. doi: 10.3390/cells14171311 (PMC12427644; doi:10.3390/cells14171311)
Supplement: Supplementary file 1 [file cells-14-01311-s001.zip › Table S6.pdf]

Table S6A. Gene Ontology (GO) of biological process: terms for SCC9-3R downregulated genes.

GOID (Biological Process)	GO Term (Biological Process)	% Associated Genes	Associated Genes	Term p-value corrected with Benjamini-Hochberg	Fold Enrichment
GO:0098609	cell-cell adhesion	4,75	DSP, LAMA5, CSTA, TENM2, ITGB4, LAMA3, ITGB2, CELSR2, PKHD1, BCAM, TRIM29, PERP, CNTN1, FAT2, ADAM8, ITGA6, STXBP6, DSG3, PKP3, CD24, DSC3, NECTIN1, MPZL2	4.36E-9	4.701735121976485
GO:0007155	cell adhesion	7,43	DDR1, SRPX, ITGB4, LAMA4, NTM, LAMA3, ITGB2, CLDN1, EFNB1, EFS, PDZD2, CCN6, CLCA2, HES1, PCDH1, MUC16, LAMB3, DSCAM, PCDH7, PCDHA10, MTSS1, FERMT1, BCAM, CEACAM1, FEZ1, MMRN2, LY6D, ANOS1, CNTN1, CLDN16, CD24, CDH16, JCAD, CD22, DSC3, NECTIN1	9.70E-9	3.060417950054495
GO:0008544	epidermis development	2,89	DSP, COL17A1, CRABP2, LAMB3, LAMA3, KLK5, GRHL3, KRT5, KRT9, NTF4, GJB5, KRT14, SPRR1B, SPRR2D	3.84E-8	7.503829648541475
GO:0022407	regulation of cell-cell adhesion	1,23	PKHD1, ADAM8, CD24, CELSR2, EPHB3, WNT4	4.09E-6	21.975501113585747
GO:0043616	keratinocyte proliferation	1,44	SDR16C5, FERMT1, FST, SFN, IRF6, KLK8, TP63	2.61E-5	11.394704281118536
GO:0007267	cell-cell signaling	3,71	FGFBP1, GDF15, ITGB2, IL18, IL36G, FGF5, EFNB1, CXCL11, BMP2, GJB4, IL1B, GJB6, CCN6, GJB5, LTB, PCDH1, S100A9, FGFR3	2.69E-5	3.380846325167038

GO:0045071	negative regulation of viral genome replication	1,65	BST2, IFITM1, TRIM6, RSAD2, OAS1, SLPI, OAS2, MX1	7.38E-5	7.643652561247216
GO:0007219	Notch signaling pathway	2,47	JAG2, NOTCH3, CEBPA, KRT19, BMP2, PERP, NEURL1, CNTN1, HES1, DTX4, DLL1, TP63	1.14E-4	4.287902656309414
GO:0061436	establishment of skin barrier	1,44	IL18, SFN, GRHL3, LSR, ABCA12, CLDN1, TP63	1.44E-4	8.546028210838902
GO:0010951	negative regulation of endopeptidase activity	1,65	SERPINB3, SERPINB4, SERPINB1, SERPINB13, SERPINB2, TIMP3, SERPINB9, SERPINB7	1.44E-4	6.894274859164155
GO:0030155	regulation of cell adhesion	1,65	PKHD1, JAG2, LAMA5, LAMA4, LAMA3, IL18, DLL1, FUT1	3.65E-4	5.959457929107999
GO:0002934	desmosome organization	8,26	DSP, PERP, PKP3, NECTIN1	3.80E-4	25.114858415526566
GO:0045087	innate immune response	5,37	ENDOD1, PRDM1, WFDC2, TRIM6, TRIM29, RNASE7, NLRP3, SLC15A3, TRIM22, GBP6, RSAD2, MX1, DAB2IP, IL36G, BST2, MARCO, OAS1, NLRP10, IFI27, SLPI, OAS2, HOOK1, S100A9, LGR4, S100A8, APOBEC3B	5.88E-4	2.1319516005717514
GO:0007389	pattern specification process	1,23	TBX1, SMO, EYA1, MFNG, FST, GRHL3	0.001	7.53445752465797
GO:0006915	apoptotic process	5,57	BEX2, IL24, ITGB2, CTSS, NHLH2, GRK5, ADAMTSL4, RASSF5, PLAGL1, RASSF6, NLRP3, DMD, RNF152, ZNF385A, TP63, UNC5B, IGFBP3, MX1, WNT7A, IL1A, SMO, IFI27, IL1B, FAS, TNFRSF25, S100A9, S100A8	0.001	2.0011417540196126
GO:0007156	homophilic cell adhesion via plasma membrane adhesion molecules	2,68	DSCAM, PCDH7, AMIGO1, CELSR2, PCDHA10, CEACAM1, FAT2, DSG3, PCDH1, CDH16, DSC3, NECTIN1, MPZL2	0.001	3.055417267129569

GO:2001235	positive regulation of apoptotic signaling pathway	1,23	DAB2IP, FAS, TPD52L1, CTSS, TP63, NFATC4	0.001	7.325167037861916
GO:0043627	response to estrogen	1,44	HOXA10, KRT19, GAL, CITED4, WNT7A, CD24, ESR1	0.001	5.493875278396437
GO:0030855	epithelial cell differentiation	1,85	TBX1, SLC9A2, FN3K, KRT19, EHF, UPK2, KRT14, KRT13, KRT9	0.001	4.0363165310667695
GO:0042475	odontogenesis of dentin-containing tooth	1,44	JAG2, TBX1, LAMA5, BMP2, SMO, FST, TP63	0.002	5.214525687969499
GO:0007165	signal transduction	9,29	CAMK2B, BEX2, IL1RN, TENM2, CRABP2, IL24, PDE3B, CBLC, NDRG2, RASIP1, PITPNC1, CXCL16, FAM83E, RASSF10, GRK5, RASSF5, CCN6, RASSF6, NLRP3, SFN, SRGAP3, FAM83B, EDARADD, CHRNB1, FGFBP1, GDF15, TNFSF15, MX1, ARAP2, MCC, ARHGAP25, ANK1, ESR1, MAPK10, BCAM, CXCL11, CEACAM1, CLEC2B, ARTN, IL1B, FAS, TNFRSF25, LTB, ASB2, RAPGEF3	0.002	1.5949960485667074
GO:0001525	angiogenesis	3,09	VAV3, TBX1, UNC5B, PDE3B, DAB2IP, IL18, WNT7A, RORA, SAT1, RASIP1, CEACAM1, ADAM8, AMOTL1, RAPGEF3, EPHB3	0.002	2.5652335930255736
GO:0032755	positive regulation of interleukin-6 production	1,85	IL1A, CD74, NLRP10, IL1B, TRPV4, PTAFR, MBP, RAB7B, IL6R	0.002	3.840378835383917
GO:0035589	G protein-coupled purinergic nucleotide receptor signaling pathway	8,26	P2RY6, P2RY2, GPR87, PTAFR	0.002	13.523385300668151
GO:0007411	axon guidance	2,68	NOTCH3, TENM2, DSCAM, PAX6, SEMA3F, EFNB1, ARTN, FEZ1, ANOS1, CNTN1, EPHB3, MATN2, NECTIN1	0.002	2.746937639198218

GO:0010628	positive regulation of gene expression	4,75	TBX1, CD74, CEBPA, EPHX2, IL36G, WNT7A, TREM2, FBLN1, PAX6, GRHL3, PRDM1, DLL1, FGF5, ACTA2, IL1A, BMP2, SMO, DNAJA4, IL1B, LAMP3, CNTN1, KPNA7, HES1	0.003	1.985998136001855
GO:0034341	response to type II interferon	1,03	BST2, CD74, IFITM1, SNCA, CXCL16	0.003	7.848393254852051
GO:0002523	leukocyte migration involved in inflammatory response	8,26	ITGB2, ADAM8, S100A9, S100A8	0.003	12.557429207763283
GO:0009887	animal organ morphogenesis	2,06	ETV7, FGF5, LAMA5, BMP2, ABLIM1, LAMB3, LAMA4, LAMA3, PAX6, FLI1	0.003	3.279925539341156
GO:0050729	positive regulation of inflammatory response	1,85	CEBPA, NLRP10, IL1B, TRPV4, IL18, NLRP3, S100A9, S100A8, SNCA	0.003	3.595991091314031
GO:0009615	response to virus	1,85	ACTA2, BST2, IFITM1, RSAD2, OAS1, OAS2, MX1, IRAK3, TRIM22	0.003	3.595991091314031
GO:0007229	integrin-mediated signaling pathway	1,85	VAV3, LAMA5, FERMT1, CEACAM1, FYB1, ITGB4, ITGB2, ADAM8, ITGA6	0.003	3.595991091314031
GO:0043410	positive regulation of MAPK cascade	2,47	TBX1, FGF5, CD74, BMP2, GDF15, IGFBP3, DAB2IP, ADAM8, IL6R, FGFR3, JCAD, WNT4	0.003	2.805383120883287
GO:0030216	keratinocyte differentiation	1,44	DSP, CSTA, KRT14, PAX6, IRF6, TP63, SPRR1B	0.004	4.591895755077619
GO:0032496	response to lipopolysaccharide	2,06	TRIM6, MAOB, SLPI, IL1B, GJB6, IRAK3, CLDN1, S100A9, S100A8, SNCA	0.004	3.1619426062713303
GO:0048488	synaptic vesicle endocytosis	1,23	NLGN4Y, NLGN4X, SNCG, STON2, SYT8, SNCA	0.0045	5.493875278396437
GO:0008285	negative regulation of cell population proliferation	4,13	DDR1, CEBPA, IFITM1, BTG2, FGFBP1, IGFBP3, IL24, DAB2IP, DLL1, IL1A, BMP2, SFRP1, CLMN, IL1B, GJB6, MMRN2, CCN6, NEURL1, IRF6, SOX7	0.005	2.0253918077037554

GO:0070269	pyroptotic inflammatory response	1,03	GSDMC, GSDMA, IFI27, NLRP3, TREM2	0.006	6.65924276169265
GO:0097191	extrinsic apoptotic signaling pathway	1,23	MLLT11, SFRP1, IFI27, FAS, G0S2, IL6R	0.006	4.975585157792999
GO:0035633	maintenance of blood-brain barrier	1,03	GJB6, MBP, DMD, LSR, CLDN1	0.007	6.463382680466396
GO:0045109	intermediate filament organization	1,44	DSP, KRT19, KRT14, KRT13, KRT5, KRT9, KRT6A	0.007	4.102093541202673
GO:0031581	hemidesmosome assembly	6,19	COL17A1, ITGB4, LAMA3	0.007	21.975501113585747
GO:0010976	positive regulation of neuron projection development	1,85	CAMK2B, DDR1, FEZ1, AMIGO1, DAB2IP, CNTN1, ITGA6, DMD, S100A9	0.007	3.1899920971334152
GO:0006954	inflammatory response	3,92	AFAP1L2, ITGB2, PTAFR, DAB2IP, LY75, IL18, NFATC4, IL1A, CXCL11, BMP2, NLRP10, HOOK1, IL1B, OLR1, NLRP3, ADAM8, DMD, S100A9, S100A8	0.008	1.9788365931664889
GO:0051607	defense response to virus	2,68	RTP4, IFITM1, CXADR, RSAD2, MX1, DDX60L, BST2, IFI27, OAS1, OAS2, NLRP3, TRIM22, APOBEC3B	0.008	2.400684995601804
GO:0031424	keratinization	1,44	IL1A, SFN, KRT5, ABCA12, SPRR1B, KRT6A, SPRR2D	0.008	3.944320712694878
GO:0070372	regulation of ERK1 and ERK2 cascade	1,03	PKHD1, CEACAM1, FAM83E, IL1B, FAM83B	0.008	6.104305864884929
GO:0046330	positive regulation of JNK cascade	1,65	IL1A, SH3RF2, CRACR2A, IL1B, TRPV4, DAB2IP, WNT7A, TPD52L1	0.009	3.413670075896815
GO:0043407	negative regulation of MAP kinase activity	1,03	BMP2, IL1B, DAB2IP, CBLC, IRAK3	0.009	5.939324625293445
GO:0008585	female gonad development	8,26	SFRP1, FST, CSMD1, WNT4	0.009	8.790200445434298
GO:2000647	negative regulation of stem cell proliferation	8,26	FERMT1, FBLN1, SFN, IRF6	0.009	8.790200445434298

GO:0070106	interleukin-27-mediated signaling pathway	6,19	OAS1, OAS2, MX1	0.010	18.836143811644924
GO:0035425	autocrine signaling	6,19	SERPINB3, S100A9, S100A8	0.010	18.836143811644924
GO:0001933	negative regulation of protein phosphorylation	1,44	NIBAN1, SMO, IGFBP3, FBLN1, PAX6, PTPN13, XDH	0.010	3.7982347603728455
GO:1902808	positive regulation of cell cycle G1/S phase transition	8,26	TBX1, FAM83E, TP63, FAM83B	0.011	8.37161947184219
GO:0030593	neutrophil chemotaxis	1,44	VAV3, CXCL11, CXADR, IL1B, ITGB2, S100A9, S100A8	0.011	3.7067110312072344
GO:0043588	skin development	1,03	RYR1, DSP, FRAS1, LTB, KRT9	0.011	5.634743875278397
GO:0016477	cell migration	2,89	VAV3, LAMA5, LAMB3, ITGB4, LAMA3, ITGB2, SHROOM2, SORL1, LIMA1, CEACAM1, EFS, HES1, CD24, EPHB3	0.012	2.1897296483288287
GO:0042102	positive regulation of T cell proliferation	1,23	CCDC88B, EFNB1, IL1B, HES1, VTCN1, ZP4	0.012	4.323049399393917
GO:0051092	positive regulation of NF-kappaB transcription factor activity	1,85	TRIM6, IL1B, IL18, NLRP3, IRAK3, RAB7B, S100A9, S100A8, TRIM22	0.013	2.887292117113456
GO:0010466	negative regulation of peptidase activity	6,19	SERPINB3, SERPINB4, CSTA	0.013	16.48162583518931
GO:0032006	regulation of TOR signaling	8,26	PKHD1, FAM83E, TREM2, FAM83B	0.014	7.643652561247216
GO:0007405	neuroblast proliferation	1,03	BTG2, SMO, ARTN, LHX5, PAX6	0.014	5.232262169901368
GO:0016055	Wnt signaling pathway	2,06	RNF43, HHEX, GRK5, AMOTL1, CD24, MCC, CPZ, NDRG2, CELSR2, LGR4	0.015	2.60065101935926
GO:0050768	negative regulation of neurogenesis	8,26	IL1B, WNT7A, PAX6, SORL1	0.016	7.325167037861915
GO:0006911	phagocytosis, engulfment	8,26	MARCO, ITGB2, TREM2, ARHGAP25	0.016	7.325167037861915
GO:0090129	positive regulation of synapse maturation	6,19	CAMK2B, NEURL1, DAB2IP	0.016	14.650334075723832

GO:0006688	glycosphingolipid biosynthetic process	8,26	B3GALNT1, A4GALT, FUT1, ST6GALNAC5	0.018	7.03216035634743 8
GO:0048844	artery morphogenesis	8,26	TBX1, NOTCH3, HES1, PRDM1	0.018	7.03216035634743 8
GO:0001755	neural crest cell migration	1,03	TBX1, EFNB1, SMO, PAX6, SEMA3F	0.018	4.883444691907943
GO:0034121	regulation of toll-like receptor signaling pathway	6,19	ESR1, S100A9, S100A8	0.020	13.18530066815144 9
GO:0003009	skeletal muscle contraction	8,26	TNNT3, TNNC2, TNNI2, RCSD1	0.020	6.761692650334075 5
GO:0043524	negative regulation of neuron apoptotic process	1,85	FOXQ1, NTF4, BTG2, UNC5B, CBLC, ADAM8, RETREG1, NFATC4, SNCA	0.022	2.619596159235387
GO:0001954	positive regulation of cell-matrix adhesion	8,26	FERMT1, SFRP1, DMD, FUT1	0.022	6.511259589210590 4
GO:0021762	substantia nigra development	1,03	MAOB, PADI2, MBP, CKB, NDRG2	0.023	4.578229398663697
GO:0009952	anterior/posterior pattern specification	1,44	TBX1, HOXA10, BTG2, HHEX, SMO, HES1, HES2	0.024	3.139357301940820 7
GO:0002064	epithelial cell development	6,19	ADAMTSL4, ESR1, TP63	0.024	11.98663697104677
GO:0019221	cytokine-mediated signaling pathway	1,85	IL1A, DUOX1, CEBPA, IL1B, IL18, IL36G, IRAK3, IL17RD, IL6R	0.025	2.551993677706732
GO:0007200	phospholipase C-activating G protein-coupled receptor signaling pathway	1,44	ANO1, P2RY6, LPAR6, P2RY2, PTAFR, PLCH2, ESR1	0.026	3.076570155902004 4
GO:0045747	positive regulation of Notch signaling pathway	1,03	JAG2, MFNG, HES1, DLL1, TP63	0.028	4.308921786977597 5
GO:0043589	skin morphogenesis	6,19	ITGB4, ITGA6, TP63	0.029	10.98775055679287 3
GO:0035455	response to interferon-alpha	6,19	BST2, IFITM1, LAMP3	0.029	10.98775055679287 3
GO:0030948	negative regulation of vascular endothelial growth	6,19	HHEX, MMRN2, DAB2IP	0.029	10.98775055679287 3

	factor receptor signaling pathway				
GO:0045216	cell-cell junction organization	8,26	PKHD1, CXADR, NLGN4X, CLDN1	0.029	5.860133630289532
GO:0050830	defense response to Gram-positive bacterium	1,65	GBP6, IL1B, RNASE7, IL18, NLRP3, IL6R, GBP4, KRT6A	0.030	2.6840306703616177
GO:0051896	regulation of phosphatidylinositol 3-kinase/protein kinase B signal transduction	8,26	CEACAM1, PDE3B, PTPN13, RAPGEF3	0.032	5.671097061570515
GO:0043065	positive regulation of apoptotic process	2,89	IGFBP3, DAB2IP, MLLT11, BMP2, SFRP1, GAL, ACVR1C, ADAMTSL4, RASSF6, NEURL1, FAS, ITGA6, PHLDA3, SNCA	0.033	1.9109131403118043
GO:0045669	positive regulation of osteoblast differentiation	1,23	CEBPA, IFITM1, BMP2, IL6R, TP63, WNT4	0.033	3.338050802063658
GO:0035493	SNARE complex assembly	6,19	CLTRN, STXBP6, SNCA	0.033	10.142538975501113
GO:0010669	epithelial structure maintenance	6,19	CXADR, LSR, PKP3	0.033	10.142538975501113
GO:0043030	regulation of macrophage activation	6,19	CD74, RORA, SNCA	0.033	10.142538975501113
GO:0035176	social behavior	1,03	TBX1, NLGN4Y, OXTR, NLGN4X, TREM2	0.036	3.99554565701559
GO:0050680	negative regulation of epithelial cell proliferation	1,03	SFRP1, DAB2IP, PAX6, MCC, MTSS1	0.036	3.99554565701559
GO:0042491	inner ear auditory receptor cell differentiation	6,19	MCOLN3, HES1, DLL1	0.039	9.418071905822462
GO:0099054	presynapse assembly	6,19	NLGN4Y, NLGN4X, WNT7A	0.039	9.418071905822462
GO:0006955	immune response	3,92	GBP6, IL1RN, FYB1, TNFSF15, PTAFR, LY75, IL18, SERPINB9, CTSS, IL1A, SLPI,	0.040	1.6536020639925906

			FCGRT, IL1B, FAS, MBP, LTB, CD22, TRIM22, NECTIN1		
GO:0031069	hair follicle morphogenesis	8,26	FOXQ1, SMO, FST, TP63	0.041	5.170706144373116
GO:0071639	positive regulation of monocyte chemotactic protein-1 production	6,19	OAS1, IL1B, TRPV4	0.044	8.7902004454343
GO:0021537	telencephalon development	6,19	BMP2, OXTR, HES1	0.044	8.7902004454343
GO:1903830	magnesium ion transmembrane transport	6,19	NIPAL4, NIPAL1, CLDN16	0.044	8.7902004454343
GO:0070488	neutrophil aggregation	4,13	S100A9, S100A8	0.044	43.95100222717149
GO:0032119	sequestering of zinc ion	4,13	S100A9, S100A8	0.044	43.95100222717149
GO:0051410	detoxification of nitrogen compound	4,13	MTARC2, MTARC1	0.044	43.95100222717149
GO:0030509	BMP signaling pathway	1,23	SFRP1, BMP2, FAM83E, FST, HES1, FAM83B	0.046	3.06634899259336
GO:0097192	extrinsic apoptotic signaling pathway in absence of ligand	8,26	IL1A, EYA1, IL1B, FAS	0.047	4.883444691907943
GO:0071407	cellular response to organic cyclic compound	8,26	CEBPA, BMP2, RNASE7, ITGA6	0.047	4.883444691907943
GO:0050832	defense response to fungus	8,26	NLRP10, RNASE7, S100A9, S100A8	0.047	4.883444691907943
GO:0043542	endothelial cell migration	8,26	S100A2, PLEKHG5, ITGB2, S100A9	0.047	4.883444691907943
GO:0030334	regulation of cell migration	1,44	FGF5, LAMA5, CEACAM1, LAMA4, LAMA3, AMOTL1, KRT5	0.048	2.652215651639659
GO:0001736	establishment of planar polarity	6,19	VANGL2, GRHL3, TP63	0.050	8.240812917594655
GO:0090162	establishment of epithelial cell polarity	6,19	FERMT1, HES1, FRMD4B	0.050	8.240812917594655
GO:0010633	negative regulation of epithelial cell migration	6,19	DAB2IP, TACSTD2, MCC	0.050	8.240812917594655

Table S6B. Gene Ontology (GO) of molecular function: terms for SCC-9R downregulated genes.

GOID (Molecular Function)	GO Term (Molecular Function)	% Associated Genes	Associated Genes	Term p-value corrected with Benjamini-Hochberg	Fold Enrichment
GO:0050839	cell adhesion molecule binding	1,85	NLGN4Y, TENM2, BCAM, CXADR, NLGN4X, ITGB2, ADAM8, PKP3, NECTIN1	8.58E-6	5.129425230970486
GO:0005509	calcium ion binding	7,02	RYR1, NOTCH3, TENM2, CBLC, FBLN1, CELSR2, DLL1, PRRG4, DUOX1, CRACR2A, CALB1, PRRG2, PCDH1, SNCA, JAG2, S100A2, GCA, PCDH7, TNNC2, SYT8, PCDHA10, VSNL1, FAT2, PADI2, TLL2, ADAM8, VWA2, DSG3, PLCH2, CDH16, S100A9, S100A8, DSC3, MATN2	9.59E-5	4.37063451632988
GO:0005102	signaling receptor binding	4,33	BEX2, TENM2, FYB1, CXADR, MAST4, TNFSF15, LAMA4, LAMA3, WNT7A, VTCN1, ABCA12, MTSS1, TRDN, ANO1, BMP2, PCSK1N, ARTN, NMU, HAP1, LTB, CD22	2.24E-4	5.478902554399244
GO:0003779	actin binding	3,51	CAMK2B, PLEKHH2, TNNC2, SHROOM2, ESPN, MTSS1, ABLIM1, CEACAM1, HOOK1, TRPV4, TNNT3, TNNI2, EPS8L1, EPS8L2, DMD, WASF3, SNCA	3.72E-4	1.9421787428463209
GO:0002020	protease binding	2,06	SERPINB3, SERPINB4, CSTA, SERPINB13, SH3PXD2A, ADAMTSL4, TIMP3, SERPINB9, MBP, ATP9A	4.71E-4	2.4251691523537753
GO:0005149	interleukin-1 receptor binding	8,26	IL1A, IL1RN, IL1B, IL36G	0.001	2.524155648368215

GO:0070273	phosphatidylinositol-4-phosphate binding	1,03	GSDMC, GSDMA, SH3PXD2A, DAB2IP, NLRP3	0.001	3.6113293673064693
GO:0005125	cytokine activity	2,47	IL1A, IL1RN, BMP2, GDF15, TNFSF15, IL1B, IL24, IL18, WNT7A, IL36G, LTB, WNT4	0.004	11.363649742457689
GO:0042803	protein homodimerization activity	5,99	CAMK2B, CEBPA, TENM2, RASIP1, HHEX, TRIM6, CLTRN, STOM, HES1, XDH, IL6R, GBP4, TRIM22, TBX1, PHETA2, NLGN4X, GCA, GDF15, EPHX2, DAB2IP, IRAK3, TPD52L1, BST2, ANO1, CEACAM1, HOOK1, CHMP4C, PADI2, NECTIN1	0.006	6.658388520971303
GO:0042834	peptidoglycan binding	8,26	HOOK1, RNASE7, NLRP3, TREM2	0.006	2.6223807097979286
GO:0043546	molybdopterin cofactor binding	6,19	MTARC2, MTARC1, XDH	0.010	1.6477292126563647
GO:0005178	integrin binding	2,06	LAMA5, FERMT1, CXADR, ITGB4, IL1B, LAMA3, CCN6, ITGB2, FBLN1, ITGA6	0.010	8.522737306843267
GO:0005200	structural constituent of cytoskeleton	1,65	DSP, KRT19, KRT14, DMD, KRT5, ANK1, KRT9, KRT6A	0.013	15.980132450331126
GO:0043565	sequence-specific DNA binding	2,89	TBX1, IRX2, EHF, TSTD1, RORA, GRHL3, ESR1, FLI1, HHEX, PLAGL1, NLRP3, HES1, IRF6, ZNF300	0.014	2.6304744774207616
GO:0043236	laminin binding	8,26	BCAM, TINAGL1, ITGA6, CTSS	0.015	3.071256687150727
GO:0097110	scaffold protein binding	1,23	DSP, NLGN4Y, NLGN4X, TREM2, KRT5, DLL1	0.017	2.0933038999264166
GO:0051015	actin filament binding	2,47	LIMA1, FERMT1, ABLIM1, PSTPIP2, CLMN, GJB6, TRPV4, SHROOM2, DMD, RCSD1, ESPN, CORO2A	0.017	7.102281089036056
GO:0036312	phosphatidylinositol 3-kinase regulatory subunit binding	6,19	DAB2IP, PTPN13, FAM83B	0.024	3.6526017029328286

GO:0045028	G protein-coupled purinergic nucleotide receptor activity	6,19	P2RY2, GPR87, PTAFR	0.024	2.1576550143907007
GO:0005515	protein binding	6,59	IFITM1, IL1RN, CBLC, KLHDC7B, ABCA12, AQP3, CLDN1, NIPAL4, ZNF607, TRIM29, RASSF5, RASSF6, CKMT1B, PTGFRN, SAMD12, SOX7, MAGEA3, TRIM22, TP63, EPHB3, ZNF165, CXADR, TMEM52B, RSAD2, UNC5B, SYTL1, KRT5, ANK1, SFRP1, BCAM, RNF128, LRATD1, IFI27, SLPI, FEZ1, RBP1, ANOS1, KCTD12, SPATA18, ANKK1, OTULINL, BEX2, MAOB, CRABP2, MAOA, RRAD, MTARC1, ENDOD1, KLK5, PHYHIPL, LYPD5, KLK8, KLK6, PKHD1, NHLH2, FAM171A1, CRACR2A, ZNF704, SLC19A3, MAP6, SFN, TSPAN1, JAG2, CDK18, PHETA2, GSDMA, FYB1, TPRG1, TNFSF15, DDIT4L, MRPL23, HSPA12A, FLI1, NFATC4, CCDC88B, BST2, MLLT11, VANGL2, GJB4, ARTN, LPAR6, GJB6, CYP2S1, NMU, GJB5, TMCC3, CD24, RETREG1, RTL8B, CD22, APOBEC3B, DDR1, SRPX, BTG2, TINAGL1, IL24, STON2, JPH1, WFDC2, TRIM6, SCML2, SH3PXD2A, ADAMTSL4, MMP28, OLR1, TIMP3, RNF152, EDARADD, VAV3, FGFBP1, SERPINB1, ELOVL4, TNNC2, IL18, ANO9, TMEM40, SERPINB9, SLC35G2, PAX6, MTSS1, ANO1, IL1A, CEACAM1, B3GNT8, GAL, VSNL1, B3GNT7, SLC7A8, IL1B, ARHGEF3, VWA2, RHOV, HAP1,	0.026	11.621914509331729

			CLDN16, RAB7B, VGLL1, RAPGEF3, MATN2, CSTA, SERPINB13, AMIGO1, PTAFR, RASIP1, NTF4, DUOX1, LIMA1, ALS2CL, RASSF10, PERP, PLAGL1, HAS3, DMD, CKB, XDH, CSMD1, RIBC2, MPZL2, CD74, GCA, GDF15, DAB2IP, KRT13, WNT7A, G0S2, PLEKHA6, GRHL3, MPP7, SH3RF2, KRT19, XK, ETNK2, CCDC8, QPCT, KRT14, FAS, LGR4, CRB3, FCMR, UPK2, RORA, PRDM1, SAT1, HOXA10, RIMS2, FCGRT, KPNA7, IL6R, SYBU, KRT6A, RNF43, CHRNB1, S100A2, DSCAM, FST, IGFBP3, LMTK3, ARMCX1, SMO, SLC9A7, DOK7, CHMP4C, LY6D, ADAM8, PKP3, S100A9, S100A8, COL17A1, NOTCH3, PARM1, NTM, TACSTD2, FBLN1, KLHL13, NDRG2, EFNB1, KLC3, ZFP57, FAM83E, CLTRN, GRK5, THEM5, NLRP3, MBP, SRGAP3, SLC15A3, RAB6B, ZNF300, FAM83B, NIBAN1, MUC16, MCC, CORO2A, CYP4F11, ESR1, BMP2, CXCL11, TRPV6, HOOK1, TRPV4, CNTN1, LHX5, AMOTL1, FGFR3, RTP4, SOWAHB, ITGB4, PDE3B, ITGB2, TREM2, PITPNC1, SDR16C5, HHEX, CALB1, HORMAD1, DSP, PLEKHG6, SHROOM2, IRAK3, TRDN, EPN3, ETV7, GFOD1, OAS1, SETBP1, SNPH, OAS2, MMRN2, ITGA6, IRF6, DSG3, CDH16, ZP4, CAMK2B, CEBPA, BLTP3A, PLEKHH2, PARD6G, ATP2C2, BARX2, DLL1, COCH,		

			PRRG4, ABLIM1, P2RY6, EFS, PRRG2, P2RY2, EPS8L1, SNCG, TNNI2, STOM, HES1, EPS8L2, LRRC8B, S1PR5, GBP4, ATP9A, SNCA, TBX1, NLGN4Y, IRX2, TCF7L1, NLGN4X, EYA1, LAMB3, CDC42BPG, MX1, SYT16, PTPN13, TPD52L1, SORL1, NAP1L5, MAPK10, CLEC2B, ZNF618, DNAJA4, NEURL1, ASB2, NECTIN1		
GO:0005543	phospholipid binding	1,65	EPN3, GRK5, PTAFR, SYT16, TREM2, SYT14, MTSS1, SNCA	0.026	11.621914509331729
GO:0005516	calmodulin binding	2,27	CAMK2B, RYR1, CEACAM1, KCNH5, TRPV6, RRAD, TRPV4, FAS, MAP6, MBP, ESR1	0.027	1.0658433436110248
GO:1990254	keratin filament binding	6,19	FAM83E, KRT14, FAM83B	0.028	2.7056308910613547
GO:0031994	insulin-like growth factor I binding	6,19	ITGB4, IGFBP3, ITGA6	0.030	2.180235125006417
GO:0008499	UDP-galactose:beta-N-acetylglucosamine beta-1,3-galactosyltransferase activity	6,19	B3GALNT1, B3GNT8, B3GNT7	0.030	10.653421633554084
GO:0005198	structural molecule activity	2,06	DSP, KRT19, LAMB3, LAMA3, KRT13, CLDN16, CLDN1, ANK1, SPRR1B, KRT9	0.035	9.83392766174223
GO:0038023	signaling receptor activity	2,27	PKHD1, NLGN4Y, NOTCH3, NLGN4X, HEPHL1, P2RY2, LY75, FAS, TREM2, TNFRSF25, MCC	0.035	9.83392766174223
GO:0005041	low-density lipoprotein particle receptor activity	6,19	OLR1, SORL1, CXCL16	0.036	2.231083064618656
GO:0004869	cysteine-type endopeptidase inhibitor activity	8,26	SERPINB3, CSTA, SERPINB13, WFDC2	0.039	2.083335786117243

GO:0030414	peptidase inhibitor activity	6,19	SERPINB1, ANOS1, A2ML1	0.046	8.522737306843267

Table S6C. Gene Ontology (GO) of cellular component: terms for SCC-9R downregulated genes.

GOID (Celllular Component)	GO Term (Cellular Component)	% Associated Genes	Associated Genes	Term p-value corrected with Benjamini-Hochberg	Fold Enrichment
GO:0005886	plasma membrane	3,98	NPFFR2, IFITM1, OXTR, IL1RN, CBLC, ABCA12, AQP3, CLDN1, NIPAL4, ZNF607, EPHB3, CXADR, UNC5B, MTUS1, SYTL1, ANK1, SFRP1, BCAM, FNDC4, FEZ1, ANOS1, RRAD, LYPD5, FAM171A1, CRACR2A, SLC19A3, STXBP6, TSPAN1, JAG2, GSDMC, AFAP1L2, GSDMA, FYB1, TNFSF15, IL36G, BST2, MARCO, VANGL2, GJB4, FXYD3, LPAR6, FAT2, PLCH2, CD22, RYR1, DDR1, TENM2, JPH1, OLR1, SERPINB3, VAV3, SERPINB4, FGFBP1, SERPINB2, SLC2A12, ANO9, SLC35G2, IL17RD, ANO1, CEACAM1, SLC7A8, RHOV, CLDN16, RAPGEF3, PTAFR, DUOX1, LIMA1, PERP, HAS3, DMD, CKB, MPZL2, CA12, CD74, GCA, DAB2IP, WNT7A, MPP7, XG, KRT19, XK, CCDC8, FAS, LGR4, CRB3, FCMR, UPK2, LY75, CELSR2, CXCL16, HEPHL1, LAMP3, TBC1D30, RGS9, IL6R, PHLDA3, RNF43, CHRNB1, KCNH5, DSCAM, PCDHA10, SLC9A2, SMO, SLC9A7, DOK7, CHMP4C, LY6D, ADAM8, PKP3, S100A9, S100A8, DSC3, COL17A1, NOTCH3, PARM1, GPR87, NTM, FUT1, EFNB1, PDZD2, CLTRN, GRK5, SLCO1B3, CLCA2,	1.57E-11	1.5305421909977555

			OR51B5, MBP, WNT4, NIBAN1, MUC16, SLC4A11, MCC, ESR1, SYT8, SLC2A9, BMP2, TRPV6, TRPV4, CNTN1, AMOTL1, FGFR3, ITGB4, ITGB2, SIRPB2, TREM2, SDR16C5, PSTPIP2, DSP, PLEKHG5, SHROOM2, LSR, IRAK3, UPK3B, TRDN, EPN3, LANCL3, TNFRSF25, ITGA6, DSG3, LTB, CDH16, ZP4, PLEKHH2, PARD6G, ATP2C2, SEMA3F, DLL1, PRRG4, P2RY6, EFS, ACVR1C, PRRG2, P2RY2, EPS8L1, STOM, EPS8L2, LRRC8B, S1PR5, PCDH1, GBP4, ATP9A, SNCA, NLGN4Y, MCOLN3, NLGN4X, PCDH7, PTPN13, SORL1, MAPK10, CLEC2B, FRAS1, TSPAN13, NEURL1, NECTIN1		
GO:0070062	extracellular exosome	1,83	DDR1, RYR1, BTG2, IL1RN, TINAGL1, UPK2, ITGB4, ITGB2, LY75, WFDC2, RIMS2, CALB1, KRT6A, GBP6, DSP, SERPINB3, SERPINB1, TMEM52B, SYTL1, SHROOM2, LSR, SERPINB9, CMBL, KRT5, KRT9, EPN3, ACTA2, ANO1, SFRP1, BCAM, CEACAM1, B3GNT8, SLPI, SMO, MMRN2, PADI2, VWA2, DSG3, IRF6, CDH16, S100A9, S100A8, RAPGEF3, LAMA5, SERPINB13, CRABP2, LAMA4, LAMA3, ENDOD1, TACSTD2, FBLN1, NDRG2, PKHD1, EFNB1, CLTRN, CREG1, EPS8L1, SNCG, STOM, EPS8L2, SFN, CKB, TSPAN1, WASF3, WNT4, NIBAN1, CYP2J2, CD74, MUC16, GCA, GDF15, EPHX2, KRT13, WNT7A,	1.79E-7	1.7479187000125997

			A2ML1, HSPA12A, PTPN13, SORL1, BST2, KRT19, FXYD3, QPCT, KRT14, CNTN1, FAT2, FAS, GALM, CD22, CRB3		
GO:0005576	extracellular region	1,71	FCMR, TINAGL1, IL24, TREM2, CTSS, CXCL16, FGF5, ADAMTSL4, CCN6, LIPG, TIMP3, OLR1, IL6R, EPHB3, SERPINB3, SERPINB1, FGFBP1, SERPINB2, CXADR, DSCAM, IGFBP3, FST, IL18, PCDHA10, IL1A, SFRP1, BCAM, GAL, GFOD1, SLPI, OAS1, IL1B, LY6D, ANOS1, PADI2, TNFRSF25, S100A9, S100A8, DSC3, MATN2, ZP4, COL17A1, LAMA5, NOTCH3, NTM, LAMA4, LAMA3, ENDOD1, KLK5, FBLN1, LYPD5, KLK8, DLL1, COCH, KLK6, NTF4, CRACR2A, PDZD2, CREG1, RNASE7, CLCA2, NLRP3, JHY, WNT4, SNCA, GCA, LAMB3, GDF15, WNT7A, IL36G, A2ML1, KLK10, MFAP5, BMP2, CXCL11, FRAS1, PRXL2A, ARTN, QPCT, NMU, TLL2, FGFR3, NECTIN1	4.88E-7	1.7518914762053455
GO:0005615	extracellular space	1,67	DDR1, IL1RN, TINAGL1, IL24, XYLT1, CPZ, WFDC2, CTSS, CXCL16, FGF5, FCGRT, CCN6, LIPG, MMP28, TIMP3, IL6R, KRT6A, SERPINB3, SERPINB4, SERPINB1, FGFBP1, SERPINB2, CXADR, IGFBP3, FST, IL18, MTUS1, SERPINB9, KRT5, FRMD4B, SERPINB7, KRT9, ACTA2, IL1A, SFRP1, GAL, FNDC4, SLPI, MFNG, IL1B, MMRN2, ANOS1, VWA2, LTB, S100A9, S100A8, COL17A1, LAMA5, CSTA, SERPINB13,	4.99E-7	1.7668626891951587

			KLK5, TACSTD2, FBLN1, SEMA3F, KLK8, KLK6, NTF4, PRRG4, PCSK1N, PRRG2, CREG1, RNASE7, STOM, SFN, CKB, XDH, WNT4, SNCA, GDF15, TNFSF15, WNT7A, IL36G, A2ML1, KLK10, SORL1, MARCO, BMP2, CXCL11, ARTN, TLL2, LGR4		
GO:0005912	adherens junction	3,92	DSP, OXTR, CXADR, LAMA3, SHROOM2, FRMD4B, DLL1, MPP7, CEACAM1, TRIM29, TRPV4, FAT2, ITGA6, STXBP6, PKP3, CDH16, JCAD, DSC3, NECTIN1	6.34E-7	4.234047093215547
GO:0005604	basement membrane	2,68	COL17A1, LAMA5, ITGB4, LAMA4, LAMA3, FBLN1, ACTA2, FRAS1, MMRN2, TIMP3, FAT2, VWA2, MATN2	1.84E-6	5.943288025039946
GO:0062023	collagen-containing extracellular matrix	5,57	COL17A1, LAMA5, SRPX, TINAGL1, LAMA4, LAMA3, FBLN1, COCH, CTSS, ADAMTSL4, MMP28, TIMP3, SERPINB1, LAMB3, GDF15, SERPINB9, MFAP5, MARCO, SFRP1, BCAM, FRAS1, SLPI, MMRN2, S100A9, S100A8, ZP4, MATN2	2.17E-6	2.9346665417759463
GO:0030054	cell junction	3,51	TENM2, CXADR, ITGB4, PLEKHG6, LSR, MPP7, FERMT1, CEACAM1, GJB4, GJB6, CLCA2, DMD, PKP3, PCDH1, TSPAN1, DSC3, CRB3	5.45E-6	4.031461107894229
GO:0009986	cell surface	7,23	SRPX, NOTCH3, IGSF3, ITGB4, ITGB2, TREM2, DUOX1, EFNB1, LAMP3, LIPG, DMD, MBP, PTGFRN, WNT4, NLGN4Y, CD74, FGFBP1, NLGN4X, KCNH5, WNT7A, SORL1, BST2, IL1A, BMP2, SFRP1, CEACAM1, TRPV4, LY6D, ANOS1, FAS, ITGA6, ADAM8, CD24, FGFR3, CD22	6.53E-6	2.3624239368938786

GO:0001533	cornified envelope	2,066	DSP, CSTA, SERPINB2, KRT14, PKP3, DSG3, SPRR1B, DSC3, KLK6, SPRR2D	7.31E-6	7.391012031139419
GO:0005911	cell-cell junction	3,09	COL17A1, TENM2, CXADR, PLEKHG5, AQP3, RASIP1, MPP7, CEACAM1, BCAM, VANGL2, PDZD2, PERP, PKP3, PCDH1, DSC3	1.26E-4	3.4465859212567245
GO:0016324	apical plasma membrane	4,75	CA12, OXTR, MAST4, UPK2, SHROOM2, PARD6G, SLC4A11, DLL1, CLDN1, PKHD1, BST2, SLC9A2, SLC2A9, ANO1, DUOX1, P2RY6, CEACAM1, VANGL2, SLC7A8, TRPV4, GJB6, IL6R, CRB3	1.68E-4	2.4877064885298537
GO:0030658	transport vesicle membrane	1,23	CD74, CEACAM1, MAP6, ABCA12, SORL1, ARFGEF3	0.002	6.187824026070213
GO:0045121	membrane raft	2,68	CXADR, UNC5B, ITGB2, CBLC, DLL1, BST2, EFNB1, HOOK1, FAS, OLR1, STOM, DMD, CD24	0.003	2.732222457008885
GO:0005923	bicellular tight junction	2,06	CXADR, SHROOM2, PARD6G, LSR, AMOTL1, CLDN16, FRMD4B, CLDN1, MPP7, CRB3	0.003	3.309408372151979
GO:0030057	desmosome	1,03	DSP, PERP, PKP3, DSG3, DSC3	0.003	7.918941461935093
GO:0016020	membrane	2,89	IFITM1, OXTR, FCMR, LY75, XYLT1, ABCA12, AQP3, CLDN1, CELSR2, CXCL16, NIPAL4, FCGRT, NIPAL1, PTGFRN, IL6R, SYBU, KRT6A, CHRNB1, KCNH5, DSCAM, TMEM52B, LMTK3, KRT5, ANK1, KRT9, SLC9A2, BCAM, ARMCX2, C1ORF115, IFI27, RNF128, SLC9A7, MFNG, LY6D, ADAM8, DSC3, COL17A1, MTARC2, IGSF3, MAOA, ENDOD1, TACSTD2, FBLN1, NDRG2, FUT1, KIAA0040, CRACR2A,	0.003	1.229883143058065

			FAM83E, SLC19A3, NLRP3, OR51B5, STXBP6, TSPAN1, FAM83B, NIBAN1, CYP2J2, B3GALNT1, GSDMC, GSDMA, MUC16, TNFSF15, ESR1, SYT8, SLC2A9, CCDC88B, BST2, MARCO, LPAR6, FXYD3, CYP2S1, TRPV4, CNTN1, TMCC3, FAT2, CD24, RETREG1, FGFR3, CD22, DDR1, RTP4, SRPX, TENM2, PDE3B, ITGB2, TREM2, OLR1, A4GALT, SUN3, SERPINB1, TMEM255A, SLC2A12, TMEM40, SERPINB9, PAX6, UPK3B, IL17RD, SORCS2, TRDN, CEACAM1, VSNL1, OAS1, SLC7A8, SNPH, OAS2, CLDN16, LTB, RAPGEF3, ST6GALNAC5, SERPINB13, LAMA4, AMIGO1, LAMA3, PTAFR, ATP2C2, DLL1, PRRG4, CLMN, HAS3, STOM, CYYR1, LRRC8B, S1PR5, CSMD1, SNCA, CA12, NLGN4Y, CD74, NLGN4X, MX1, DAB2IP, SYT16, KIAA1549L, SYT14, SORL1, XG, FRAS1, XK, TSPAN13, DNAJA4, NECTIN1		
GO:0005610	laminin-5 complex	6,19	LAMA5, LAMB3, LAMA3	0.007	22.173036093418258
GO:0030175	filopodium	1,44	ACTA2, TENM2, CXADR, TRPV4, ITGA6, DMD, RAPGEF3	0.009	3.8323766087389584
GO:0030056	hemidesmosome	6,19	COL17A1, ITGB4, LAMA3	0.012	16.629777070063696
GO:0005856	cytoskeleton	4,33	EVC2, PLEKHH2, CDC42BPG, SHROOM2, KRT13, FRMD4B, PTPN13, ANK1, KRT9, ACTA2, FERMT1, KRT19, RNF128, KRT14,	0.013	1.7978137373041831

			STOM, DMD, HAP1, S100A9, S100A8, WASF3, MPZL2		
GO:0005861	troponin complex	6,19	TNNT3, TNNC2, TNNI2	0.016	14.782024062278841
GO:0000139	Golgi membrane	5,37	NOTCH3, ST6GALNAC2, PARM1, XYLT1, ATP2C2, ABCA12, FUT1, CRACR2A, NIPAL1, HAS3, NLRP3, GFY, A4GALT, RAB6B, SYBU, GBP4, B3GALNT1, CD74, LMTK3, IL17RD, SORL1, B3GNT8, B3GNT7, SLC9A7, MFNG, ST6GALNAC5	0.018	1.6239406716306333
GO:0016323	basolateral plasma membrane	2,68	CA12, DSP, CXADR, SLC4A11, ATP2C2, AQP3, CLDN1, ANK1, SLC2A9, P2RY6, SLC7A8, SLCO1B3, CDH16	0.019	2.1431187302188652
GO:0014701	junctional sarcoplasmic reticulum membrane	6,19	RYR1, JPH1, TRDN	0.020	13.303821656050957
GO:0030141	secretory granule	1,44	PCSK1N, GAL, IL1B, KLK5, KLK8, KLK10, KLK6	0.021	3.233567763623496
GO:0009925	basal plasma membrane	1,23	CEACAM1, SLC7A8, ITGB4, SLCO1B3, CLCA2, TACSTD2	0.023	3.6955060155697104
GO:0070821	tertiary granule membrane	1,23	CEACAM1, ITGB2, PTAFR, STOM, OLR1, ADAM8	0.024	3.6448826454934125
GO:0005794	Golgi apparatus	7,43	TENM2, EHF, PARM1, PDE3B, NDRG2, BARX2, FUT1, PKHD1, NIPAL1, PERP, PLAGL1, LIPG, HAS3, PTGFRN, RAB6B, ARFGEF3, GBP4, TRIM22, RSAD2, GDF15, MTUS1, SLC35G2, SERPINB9, IL17RD, ESR1, SORL1, CCDC88B, BST2, SMO, RNF128, SLPI, FEZ1, FAT2, RAB7B, FGFR3, ST6GALNAC5	0.025	1.4513259988419223

GO:0016328	lateral plasma membrane	1,44	CEACAM1, VANGL2, SLC7A8, TACSTD2, DMD, CLDN1, MPP7	0.025	3.1042250530785562
GO:0030666	endocytic vesicle membrane	1,23	CAMK2B, CD74, MARCO, SMO, WNT7A, WNT4	0.025	3.5956274746083663
GO:0016529	sarcoplasmic reticulum	1,03	RYR1, JPH1, XDH, ANK1, TRDN	0.027	4.347654135964365
GO:0097731	9+0 non-motile cilium	6,19	PKHD1, C1ORF115, SMO	0.028	11.086518046709129
GO:0034993	meiotic nuclear membrane microtubule tethering complex	6,19	SUN3, CLMN, DMD	0.028	11.086518046709129
GO:0045095	keratin filament	1,44	FAM83E, KRT14, KRT13, KRT5, FAM83B, KRT9, KRT6A	0.028	3.0138107311442295
GO:0030426	growth cone	1,65	TENM2, CXADR, DSCAM, FEZ1, TRPV4, HAP1, NDRG2, SNCA	0.035	2.589551660545198
GO:0009898	cytoplasmic side of plasma membrane	1,44	EPN3, NLRP10, CHMP4C, ATP2C2, SAMD12, ANK1, MTSS1	0.042	2.7471018168836783
GO:1990660	calprotectin complex	4,13	S100A9, S100A8	0.044	44.3460721868365 2
GO:0031982	vesicle	1,85	SERPINB3, CRACR2A, MUC16, LAMP3, EPS8L1, STOM, EPS8L2, TSPAN1, SYBU	0.049	2.2296907803437356

Table S6D. KEGG terms for the dataset of differentially expressed genes comparing SCC-9P with SCC-9R cell lines: terms for SCC-9R downregulated genes.

KEGGID	KEGG Term	% Associated Genes	Associated Genes	Term p-value corrected with Benjamini-Hochberg	Fold Enrichment
hsa05146	Amoebiasis	2,27	SERPINB3, LAMA5, SERPINB4, SERPINB13, LAMB3, IL1B, LAMA4, LAMA3, ITGB2, SERPINB9, RAB7B	1,71E+12	4,43229E+15
hsa05200	Pathways in cancer	5,16	CAMK2B, LAMA5, CEBPA, NOTCH3, LAMA4, LAMA3, DLL1, FGF5, RASSF5, HES1, IL6R, WNT4, JAG2, TCF7L1, LAMB3, PLEKHG5, WNT7A, ESR1, MAPK10, BMP2, SMO, LPAR6, FAS, ITGA6, FGFR3	2,08E+15	1,94664E+16
hsa04060	Cytokine-cytokine receptor interaction	3,30	IL1RN, GDF15, TNFSF15, IL24, IL18, IL36G, CXCL16, IL1A, CXCL11, BMP2, ACVR1C, IL1B, FAS, TNFRSF25, LTB, IL6R	5,31E+15	2,22831E+16
hsa05165	Human papillomavirus infection	3,51	LAMA5, NOTCH3, TCF7L1, LAMB3, ITGB4, LAMA4, LAMA3, MX1, PARD6G, WNT7A, MFNG, FAS, ITGA6, HES1, HES2, CRB3, WNT4	6,30E+15	2,11874E+15
hsa00330	Arginine and proline metabolism	1,23	MAOB, MAOA, CKMT1B, CKB, SAT1, AGMAT	6,67E+16	4,98028E+16
hsa04310	Wnt signaling pathway	2,27	CAMK2B, MAPK10, RNF43, SFRP1, TCF7L1, VANGL2, WNT7A, MCC, LGR4, NFATC4, WNT4	9,03E+15	2,62371E+16
hsa04360	Axon guidance	2,27	CAMK2B, EFNB1, ABLIM1, SMO, UNC5B, PARD6G, SEMA3F, SRGAP3, EPHB3, NFATC4, WNT4	1,30E+16	2,48112E+15

hsa04514	Cell adhesion molecules	2,06	NLGN4Y, NLGN4X, ITGB2, CNTN1, ITGA6, CLDN16, VTCN1, CLDN1, CD22, NECTIN1	1,319E+16	2,64346E+16
hsa04330	Notch signaling pathway	1,23	JAG2, NOTCH3, MFNG, HES1, DTX4, DLL1	1,61E+15	4,01636E+15
hsa04512	ECM-receptor interaction	1,44	LAMA5, FRAS1, LAMB3, ITGB4, LAMA4, LAMA3, ITGA6	1,98E+16	3,26423E+16
hsa05164	Influenza A	2,06	IL1A, RSAD2, OAS1, OAS2, IL1B, MX1, IL18, FAS, NLRP3, KPNA7	2,25E+16	2,41293E+16
hsa05224	Breast cancer	1,85	JAG2, FGF5, NOTCH3, TCF7L1, WNT7A, HES1, DLL1, ESR1, WNT4	2,58E+16	2,52379E+16
hsa00603	Glycosphingolipid biosynthesis - globo and isoglobo series	6,19	B3GALNT1, A4GALT, FUT1	5,50E+15	7,78169E+14
hsa05143	African trypanosomiasis	8,26	IL1B, LAMA4, IL18, FAS	5,82E+14	4,48674E+15
hsa05217	Basal cell carcinoma	1,03	BMP2, TCF7L1, SMO, WNT7A, WNT4	6,40E+15	3,29384E+15
hsa04640	Hematopoietic cell lineage	1,23	IL1A, IL1B, ITGA6, CD24, IL6R, CD22	8,90E+15	2,51529E+16
hsa04750	Inflammatory mediator regulation of TRP channels	1,23	CAMK2B, MAPK10, CYP2J2, IL1B, P2RY2, TRPV4	8,90E+15	2,51529E+16
hsa05150	Staphylococcus aureus infection	1,23	KRT19, KRT14, ITGB2, PTAFR, KRT13, KRT9	9,20E+15	2,49014E+16
