# Supplementary figures and images for: Generation and Characterization of Cisplatin-Resistant Oral Squamous Cell Carcinoma Cells Displaying an Epithelial–Mesenchymal Transition Signature
Source: Cells. 2025 Aug 24;14(17):1311. doi: 10.3390/cells14171311 (PMC12427644; doi:10.3390/cells14171311)

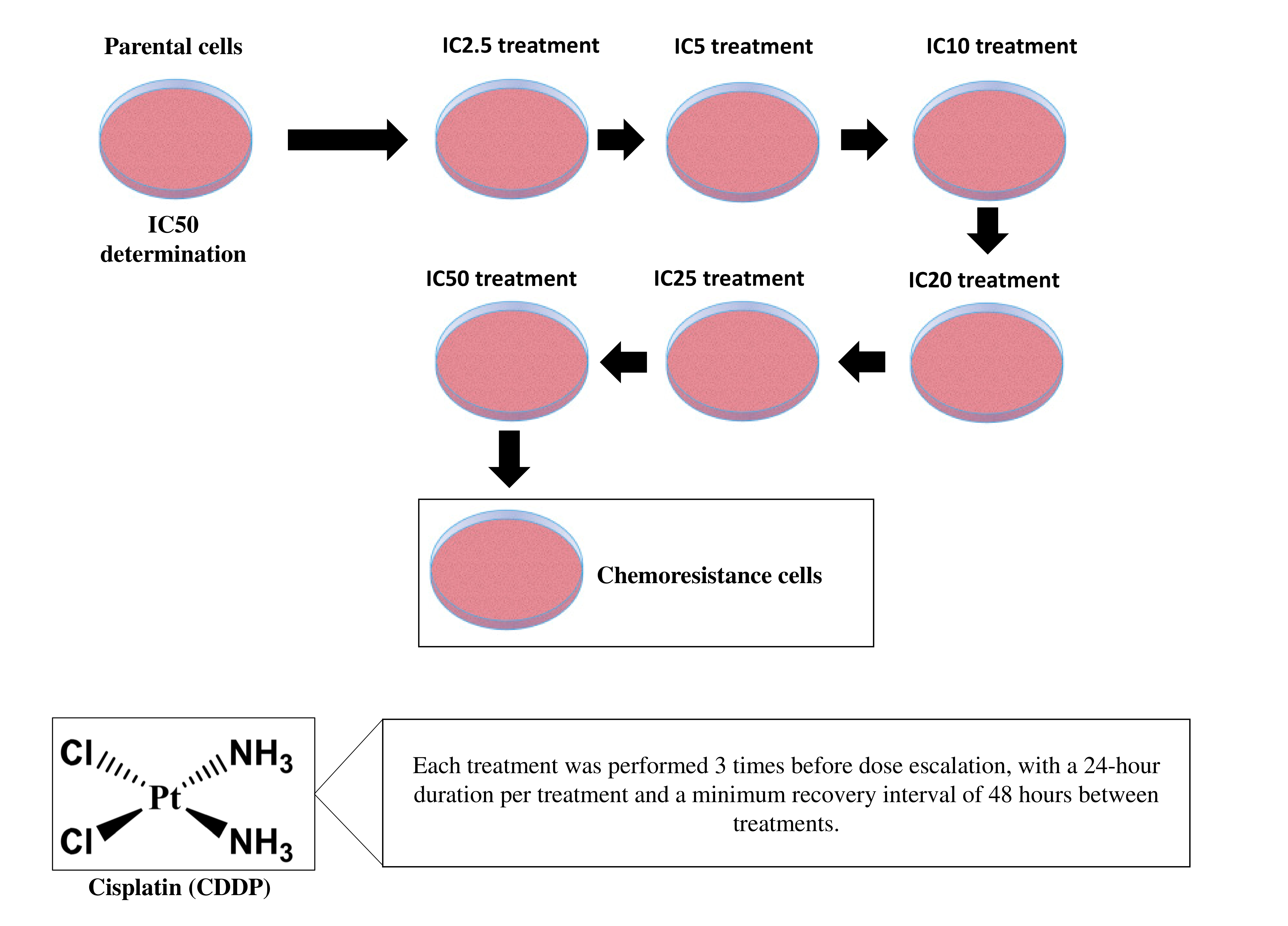

Supplement: Supplementary file 1 [file cells-14-01311-s001.zip › Figure S1.tiff]

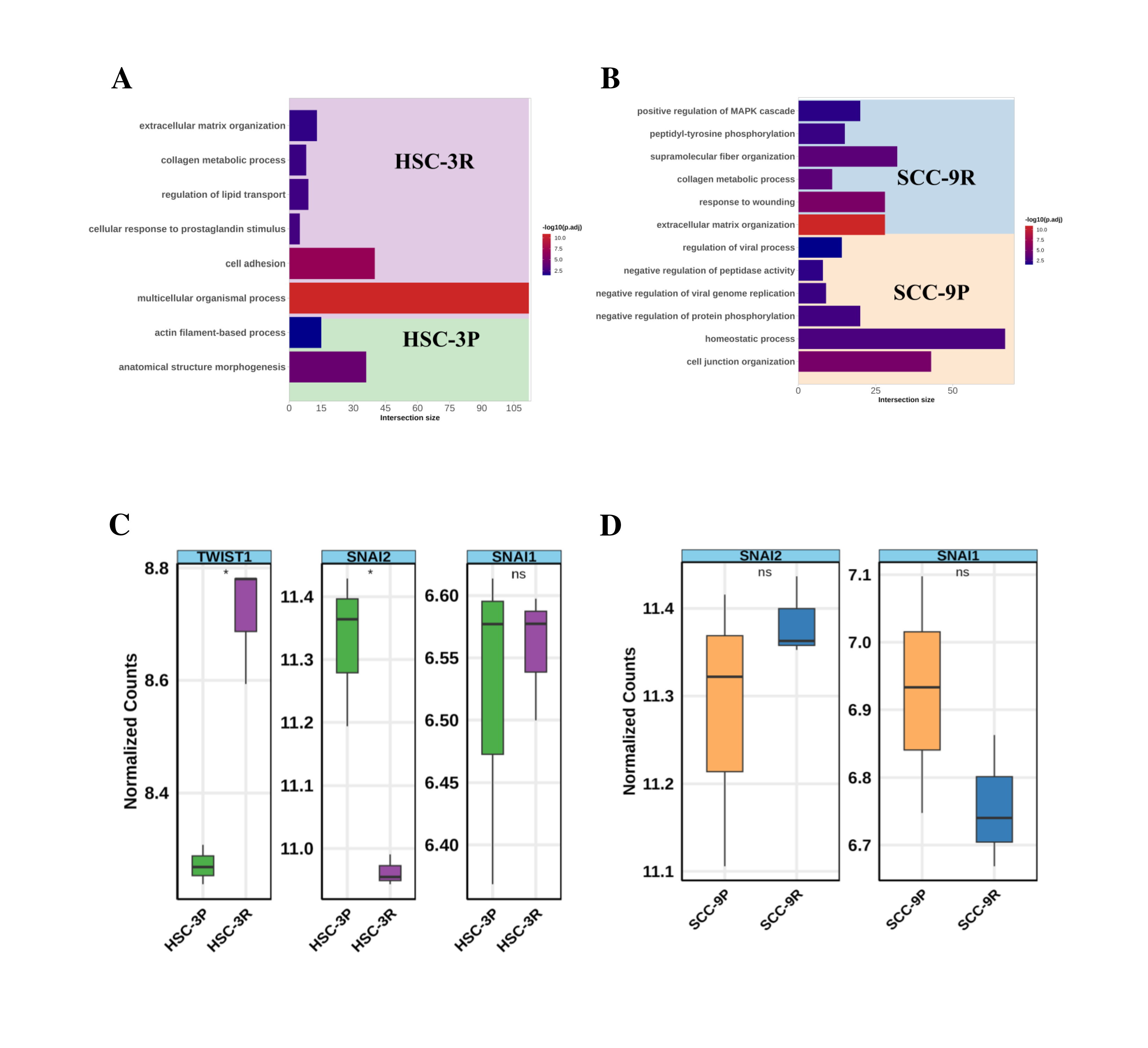

Supplement: Supplementary file 1 [file cells-14-01311-s001.zip › Figure S2.tiff]
